# Construction of a novel risk model for esophageal squamous cell carcinoma associated with purinergic signaling pathways and chemoradiotherapy sensitivity genes

**DOI:** 10.3389/fmed.2026.1746281

**Published:** 2026-03-17

**Authors:** Xiang Zhan, Fengge Zhou, Yuanhui Yang, Lingli Lei, Miao Li, Jixian Li, Alei Feng, Yan Qu, Renya Zeng, Zhe Yang

**Affiliations:** 1 Tumor Research and Therapy Center, Shandong Provincial Hospital, Shandong University, Jinan, Shandong, China; 2 Department of Radiotherapy, The Second Hospital of Hebei Medical University, Shijiazhuang, Hebei, China; 3 Tumor Research and Therapy Center, Shandong Provincial Hospital Affiliated to Shandong First Medical University, Jinan, Shandong, China; 4 Pathology Department, Shandong Provincial Hospital, Shandong University, Jinan, Shandong, China

**Keywords:** anti-tumor drug targets, chemoradiotherapy, esophageal squamous cell carcinoma, prognostic signature gene, purinergic signaling

## Abstract

**Background:**

Esophageal squamous cell carcinoma (ESCC) significantly impacts public health. Variability exists in patients’ responses to chemoradiotherapy (CRT), and the role of purinergic signaling (PS) in ESCC, related to tumor migration, remains unclear.

**Methods:**

Survival-related genes among PS-related genes were identified using univariate Cox analysis. Patients from The Cancer Genome Atlas (TCGA)-ESCC dataset were categorized based on optimal scoring thresholds. Differential expression analysis was employed to identify differentially expressed genes (DEGs) between tumors and controls and across scoring groups. Intersecting genes from DEGs were identified and further analyzed in the GSE45670 dataset to identify genes associated with CRT sensitivity, which were designated as candidate genes. A prognostic signature was developed, and gene set enrichment analysis (GSEA), mutation analysis, immune infiltration, and drug sensitivity were implemented. In addition, we used Reverse Transcription quantitative Polymerase Chain Reaction (RT-qPCR) for experimental validation to further screen potential anti-tumor drug targets. Finally, the expression of prognostic signature genes in pan-cancer were explored.

**Results:**

A total of four prognostic signature genes (ANXA10, ERICH5, HRG, and AMN) were identified. GSEA linked the risk group to pathways like cell adhesion molecules. Mutational analysis suggested a potential missense mutation in ANXA10. High-risk patients were associated with increased infiltration of plasmacytoid dendritic cell and T follicular helper cells. Drugs like VX.702 and BMS.754807 were more effective in the high-risk group. In esophageal cancer, ANXA10 was remarkably down-regulated, and HRG was up-regulated. ESCC tumor tissues exhibited markedly decreased AMN and ERICH5 expression but increased HRG compared to normal controls.

**Conclusion:**

This study identified prognostic signature genes associated with CRT and PS in ESCC and constructed a risk model that may predict patient survival, which could provide valuable insights for future research on ESCC diagnosis and treatment.

## 1 Introduction

Esophageal cancer (EC) is one of the most frequent and aggressive gastrointestinal tumors and the sixth leading cause of cancer-related death worldwide (1). Due to the lack of typical clinical symptoms and precise treatment for early-stage EC, the 5-year survival rate of most EC patients is < 20% (2). As for the histological subtypes, esophageal squamous cell carcinoma (ESCC) and esophageal adenocarcinoma are the main types, with ESCC accounting for over 85% of all occurrences (3). ESCC often invades the tissues surrounding the esophagus and metastasizes through the lymphatic system to distant organs. Smoking, alcohol consumption and chronic oesophageal disease are causative factors for ESCC (4). However, the limited research on the molecular etiology of ESCC exacerbates its poor prognosis. Nowadays, chemoradiotherapy (CRT) is the standard treatment for unresectable advanced ESCC. Although the efficacy of CRT is surprising, there are still some people who are difficult to benefit from it, and even local recurrence and distant metastasis (5). Previous studies have tried to change chemotherapy drugs and radiation doses to improve patient survival, but have not met expectations (6, 7). Due to the heterogeneity of ESCC, CRT often produces variable responses in patients. The underlying molecular mechanisms of CRT sensitivity or resistance are important to improve prognostic accuracy and elucidate potential therapeutic targets (8). Therefore, exploring new prognostic genes related to CRT sensitivity and their potential mechanisms will help to understand the pathogenesis and molecular mechanism of ESCC, and provide a new theoretical basis and target for clinical diagnosis and treatment.

Purinergic signaling (PS) is a complex network mediated by extracellular purine and pyrimidine nucleotides/nucleosides (e.g., ATP and adenosine) and their corresponding receptors. These receptors are categorized into P1 (A1, A2A, A2B, A3) and P2 families. The P2 family is further subdivided into ionotropic P2X receptors (P2X1-7) and metabotropic G protein-coupled P2Y receptors (P2Y1, 2, 4, 6, 11, 12, 13, 14) (9). PS orchestrates a broad spectrum of critical physiological and pathophysiological processes, including cell proliferation, migration, differentiation, and apoptosis, often by coordinating with other signaling systems (10). Research has revealed that PS plays a central role in the tumor microenvironment (TME). Adenosine triphosphate (ATP) released by cancer cells has been shown to promote tumor growth, angiogenesis, and potent immune suppression through autocrine and paracrine mechanisms (11). Specifically, PS drives tumor progression by reshaping the TME through distinct mechanisms across different cancer types: In the context of colorectal cancer, PS has been shown to promote immune evasion and malignant progression by directing the TME’s immune response toward an inhibitory state (12). In prostate cancer, PS has been identified as a central regulator of TME homeostasis, determining tumor progression direction by integrating metabolic, immune, and stress signaling (13). In cervical cancer, PS has been revealed to serve as a pivotal bridge linking malignant biological behavior of tumor cells with host immune defense (14). There is compelling evidence to suggest that specific purinergic pathways (e.g., P2X7 receptors) are closely associated with cancer metastasis and progression (9), thus revealing highly attractive therapeutic targets. However, the precise mechanism of PS action in ESCC remains to be elucidated. Although emerging studies suggest that PS may regulate the release of pro-metastatic factors during CRT in other malignancies (15), direct evidence linking PS to ESCC pathogenesis, CRT resistance, or patient prognosis remains scarce. This critical knowledge gap emphasizes the necessity for systematic investigation into the integrated role and mechanistic basis of PS-related genes (PSRGs) in ESCC, particularly regarding their impact on CRT response and patient survival.

In summary, this study focuses on the value of genes related to PS and CRT sensitivity on the prognosis, biological function, molecular regulatory mechanism, immune characteristics and potential therapeutic agents of ESCC by bioinformatics and experimental verification means, so as to provide improved ideas for developing reasonable and personalized treatment plans for ESCC patients.

## 2 Materials and methods

### 2.1 Data source

The Cancer Genome Atlas (TCGA)-ESCC subset was obtained from the UCSC Xena database.^1^ Based on the clinical phenotype file (primary_diagnosis.diagnoses), we selected samples with a pathological diagnosis of “ESCC” while excluding those diagnosed as esophageal adenocarcinoma. Only samples from primary tumors (code 01) and adjacent normal tissues (code 11) were retained. This process yielded a final subset comprising 80 ESCC patients with complete gene expression and survival information, along with 11 normal controls. GSE53622 and GSE45670 were retrieved from Gene Expression Omnibus (GEO) database.^2^ Of these, GSE53622 (platform: GPL18109) included 60 ESCC patients and 60 controls that were employed as validation of risk model. GSE45670 (platform: GPL570) contained pretreatment cancer biopsies from 17 CRT treatment-insensitive ESCC patients and 11 sensitive patients. Then, “WP PURINERGIC SIGNALING” was retrieved from Molecular Signatures Database (MSigDB),^3^ which contained 33 PSRGs for this study. All the aforementioned data sets are accessible to the public.

### 2.2 Differential expression analysis

The differentially expressed genes (DEGs1) were acquired between ESCC and control in TCGA-ESCC dataset with adj.*P*-value < 0.05 & | log_2_Fold Change (FC)| > 1 using DESeq2 (v1.38.0) (16). The ggplot2 (v3.4.4) and pheatmap (v1.0.12) were employed to plot volcano graph and heatmap to display the DEGs1 results, respectively (17, 18). Further, univariate Cox analysis of 33 PSRGs in TCGA-ESCC dataset was conducted using survival (v3.5-3) to screen for prognostically relevant PSRGs (Hazard Ratio (HR) ≠ 1 & *p* < 0.2) (19). Then, GSVA (v1.46.0) was employed to calculate the PSRGs scores of each ESCC sample, the optimal thresholds for the scores were found by using the surv cutpoint function of survminer (v0.4.9), and the patients were classified into various PSRGs scoring groups (20, 21). Subsequently, Kaplan-Meier (K-M) and log-rank tests were utilized to assess the difference in survival of patients between PSRGs scoring groups. Similarly, DEGs2 between the groups were acquired by difference analysis using DESeq2 with adj.*P*-value < 0.05 & | log_2_FC| > 1.

### 2.3 Enrichment analysis and ikprotein-protein interaction network

The above acquired DEGs were overlapped to access the intersection genes. Then to screen the genes among them that were associated with sensitivity and insensitivity to CRT treatment in ESCC, Wilcoxon was employed to determine the notable difference genes of the intersection genes between patients with sensitivity and insensitivity to CRT treatment in GSE45670 (*p* < 0.05), which were recorded as candidate genes. Further, enrichment analysis were implemented for candidate genes to probe pathways and functions associated with them using clusterProfiler (v4.2.2), containing Gene Ontology (GO) and Kyoto Encyclopedia of Genes and Genomes (KEGG) (*p* < 0.05) (22). To understand the interaction relationship between the corresponding proteins of these genes, a PPI network was constructed in STRING database based on an interaction score > 0.4 and visualized using Cytoscape (v3.9.1) (23).

### 2.4 Construction, evaluation and validation of risk models

A wide range of regression analyses were conducted to screen the prognostic signature genes of ESCC. Firstly, based on HR ≠ 1 & *p* < 0.1, survival was applied to candidate genes to select survival-related genes by univariate Cox analysis using survival (v3.5-3) (24). Then, genes with *p* < 0.1 and HR ≠ 1 were subjected to proportional hazards (PH) assumption test (Schoenfeld residual method, *p* > 0.05), followed by Least Absolute Shrinkage and Selection Operator (LASSO) analysis using glmnet (v4.1-2) of genes that passed the test to select prognostic signature genes conditional on the lowest error rate (25). Risk models were constructed based on the expression of genes obtained from LASSO analysis. The risk score was calculated as: $r\,i\,s\,k\,s\,c\,o\,r\,e=\sum_{i=1}^{n}\left(c\,o\,e\,f\,i*X\,i\right)$. where Xi was the relative expression of prognostic signature genes i, coefi was the LASSO Cox coeffcient of prognostic signature genes i.

The ESCC patients in TCGA-ESCC dataset were categorized into different risk cohorts according to best cut-off value of risk score. K-M curves were then used to assess the survival differences of patients between risk cohorts using survival (v3.5-3) (21). The Receiver Operating Characteristic (ROC) curves of patients at 1–3 years were plotted using survivalROC (v1.0.3), and the true positive, false positive and corresponding Area Under the Curve (AUC) values were calculated for each sample (26). To internally validate the model and correct for potential overfitting, a bootstrap resampling procedure with 1,000 replicates was conducted. In each bootstrap iteration, a model was fitted on the resampled dataset, and its apparent discriminative performance (C-index and time-dependent AUC) was evaluated on that same bootstrap sample. The model was then applied to the corresponding out-of-bag (OOB) samples to obtain a more unbiased estimate of its performance. The difference between the apparent and OOB performance was recorded as the optimism for that iteration. The average optimism across all 1,000 bootstrap replicates was subtracted from the apparent performance of the original model (trained on the full dataset) to obtain the optimism-corrected C-index and AUC values. These corrected metrics are reported as the final, internally validated estimates of the model’s prognostic performance. Additionally, the predictive accuracy and validity of the model was verified in GSE53622.

### 2.5 Stratified and independent prognostic analyses

To explore the association of clinical features (gender, age, histological, stage, T stage, N stage, M stage) subtypes with risk scores and prognostic signature genes, different clinical features in TCGA-ESCC dataset were stratified. The risk scores between different clinical subtypes and the differences of prognostic signature genes in different subtypes were examined using Wilcoxon (*p* < 0.05). Moreover, we explored the survival differences among patients with different clinical features and survival differences between risk cohorts in various clinical feature subtypes (*p* < 0.05). Next, independent prognostic analyses were utilized to assess the prognostic value of clinical features and risk scores. The univariate Cox analyses combining risk scores and clinical characteristics were performed. Then, for those factors where *p* < 0.05 and HR ≠ 1, the Schoenfeld residual method was employed to conduct a PH assumption test. Factors identified by this test were included in multifactor Cox analysis to screen for independent prognostic factors. Subsequently, a nomogram was constructed using the rms (v6.5-0) to assess the probability of survival of patients with ESCC within 1–3 years (21). Finally, the predictive accuracy and validity of the nomogram were further evaluated using calibration curves and decision curves.

### 2.6 Gene set enrichment analysis and single-cell functional analysis

To understand the pathways and functions associated with ESCC patients in different risk cohorts, GSEA was performed using clusterProfiler. Differential expression analysis was conducted between risk cohorts using DESeq2 to obtain log2FC and rank them from largest to smallest. GSEA was carried out using “c2.cp.kegg.v7.4.symbols.gmt” and “c5.go. v7.4. symbols.gmt” as background gene sets in MSigDB (*p* < 0.05). Further, single-cell functional differences in prognostic signature genes in different tumors probed in CancerSEA^4^ (| R| > 0.3, *p* < 0.05), in which CancerSEA contained 14 tumor-associated cellular functions from 41,900 cancer cells from 25 cancers.

### 2.7 Somatic mutation and immunoassay

To explore the mutation profile of genes in the risk cohort, somatic mutation information was analyzed using maftools (v2.14.0) to compare their mutational differences (27). In addition, mutations in prognostic signature genes were also investigated. Next, the immune score, stromal score, and estimate score in the risk cohort were calculated using Estimate (v1.0.13) and their distributional differences were assessed, and then their correlation with the risk score was analyzed by Spearman (| R| > 0.3 & *p* < 0.05) (28). Further, the proportion of 28 immune cells infiltrated in the risk cohort samples was calculated using single-sample GSEA (ssGSEA). The differences in their infiltration levels between risk cohorts were compared. And the correlation between prognostic signature genes and differential immune cells was investigated (| R| > 0.3 & *p* < 0.05).

### 2.8 Methylation analysis

N6-methyladenosine (m6A) is involved in the pathogenesis of a wide range of diseases, including cancer. Therefore, Sequence-based RNA Adenosine Methylation site Predictor (SRAMP)^5^ was employed to predict the m6A modification sites of the prognostic signature genes and their locations in the RNA secondary structure.

### 2.9 Drug sensitivity and molecular networks

To assess the relationship between chemotherapy response and risk cohort, 138 chemotherapy/targeted therapy drugs for ESCC were acquired at the Genomics of Drug Sensitivity in Cancer (GDSC).^6^ Then, pRRophetic (v 0.5) was employed to predict half-maximal inhibitory concentrations (IC_50_) of the therapeutic agents for each patient to infer drug sensitivity (29). To explore the molecular mechanisms played by the characterized genes in ESCC a long non-coding RNA (lncRNA)-miRNA-mRNA network was constructed. The miRDB and microcosm databases in the R package multiMiR (v 1.20.0) were used to predict the miRNAs of the feature genes, and the common miRNAs were selected as target miRNAs for subsequent analysis. Then the lncRNAs corresponding to the target miRNAs were predicted in the Starbase database.^7^ Subsequently, the lncRNA-miRNA-mRNA network was constructed using Cytoscape.

### 2.10 Expression analysis of prognostic signature genes

RNA expression data of gene expression genes from 33 tumors in TCGA as well as normal tissues of GTEx were obtained from the UCSC Xena database to explore the expression of the prognostic signature genes in these tumors. Then, the expression profiles of the prognostic signature genes were extracted in TCGA-ESCC dataset and GSE53622 to detect their expression differences between ESCC and control (*p* < 0.05).

### 2.11 Cell culture and treatment

The ECA109 and TE1 cells were purchased from the Shanghai Institute of Life Sciences at the Chinese Academy of Sciences. All cells were cultured in RPMI 1640 medium supplemented with 10% fetal bovine serum (FBS) and 1% penicillin/streptomycin, in a humidified incubator at 37°C and 5% CO*2*. Both cell lines were used at low passages ( < 20). The cells were then treated with an anti-PD-1 antibody (Pembrolizumab, 10 μg/mL), cisplatin (50 μM) or radiation therapy (2 Gy per fraction, three fractions in total).

### 2.12 Reverse transcription quantitative polymerase chain reaction

Perform reverse transcription using a complementary DNA (cDNA) synthesis kit. For RT-qPCR, amplify 2 μL of the reverse transcription reaction in a 20 μL total volume using the TB Green Premix Ex Taq II Kit, running 40 cycles of initial denaturation at 95°C for 30 s, denaturation at 95°C for 5 s and annealing at 61°C for 34 s. The relative expression level of the GAPDH target gene was determined using the 2^(−ΔΔ)Ct method. Primer sequences are shown in Table 1.

**TABLE 1:** Primer sequences for RT-qPCR analysis.

Gene	Primer sequence (5′→3′)
AMN	Forward 5′-GACCTCTGTGGAGCCGTTGTG-3′
	Reverse 5′-GACGAGCGTGGCACCTTGG-3′
HRG	Forward 5′-AGTATTCGTGTGCCGTGAGTCC-3′
	Reverse 5′-GCCATCCCGTCGCCTTTTATTG-3′
ERICH5	Forward 5′-GGCGACAGCAGCAGGTTCC-3′
	Reverse 5′-TGGTTGGGCAAAGCAGGACTC-3′
GAPDH	Forward: 5′-GTGGACCTGACCTGCCGTCTAG-3
	Reverse: 5′-GGAGTGGGTGTCGCTGTTGAAG-3

### 2.13 Statistical analysis

All analyses were performed using the R programming language (v4.2.2) and compared data from different groups using the Wilcoxon test. Unless otherwise stated, a *p* < 0.05 was considered statistically significant.

## 3 Results

### 3.1 Identification of 406 intersection genes

**FIGURE 1:**
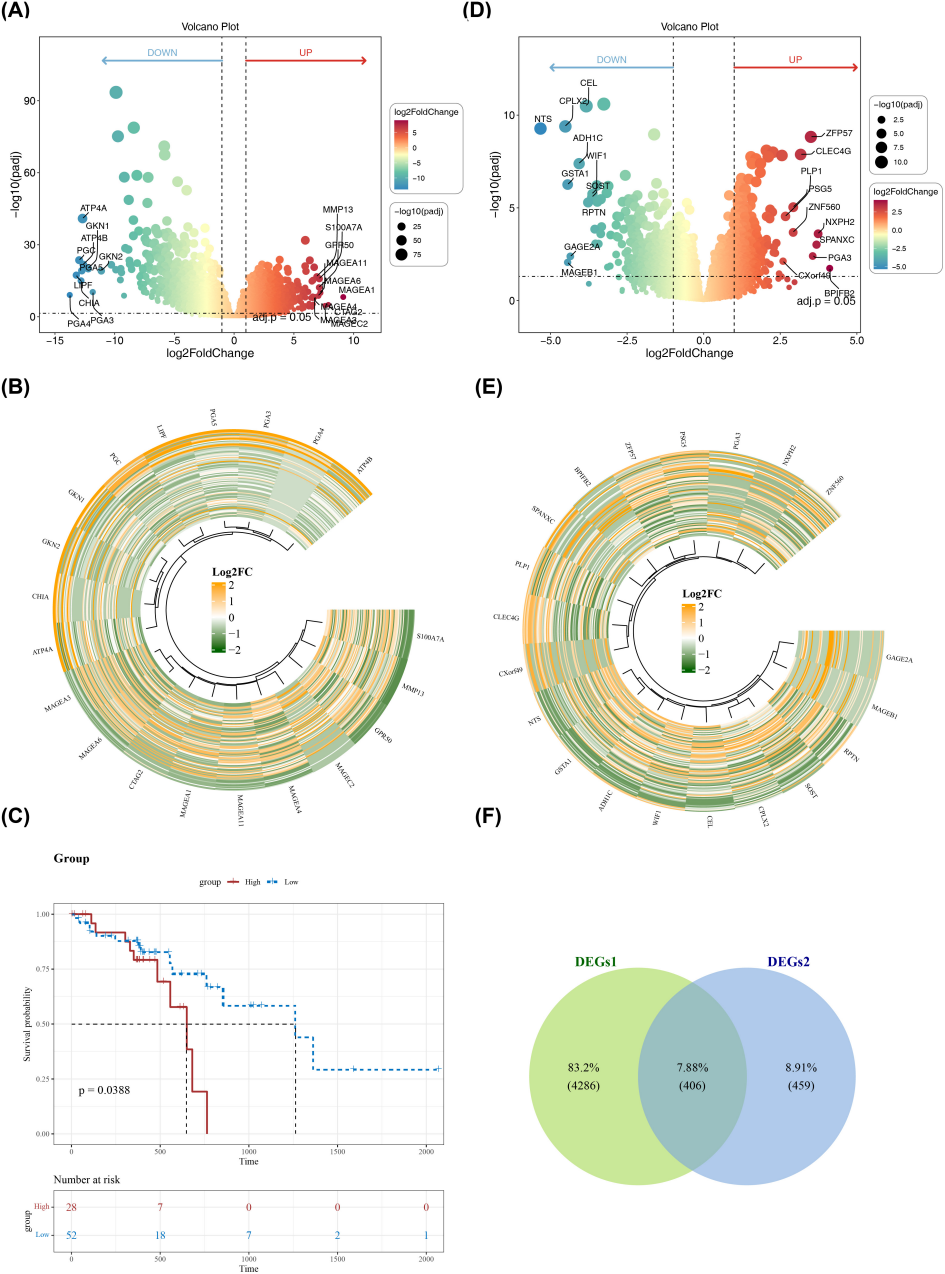
Differential gene expression analysis and identification of intersection gene. **(A,B)** Volcano and heat maps of DEGs1 in the TCGA-ESCC dataset. **(C)** The Overall survival analysis by Kaplan–Meier in high- vs. low- PSRGs scoring groups. **(D,E)** Volcano and heat maps of DEGs2 in the high- vs. low- PSRGs scoring groups. **(F)** The above DEGs were overlapped to gain intersection genes.

The 4,692 DEGs1 were gained between ESCC and control in TCGA-ESCC dataset, containing 2,211 upregulated and 2,481 downregulated genes (Figures 1A,B). Then, the 8 prognostically relevant PSRGs were selected, including GNAS, GNAI3, PANX1, ADORA2B, ADORA3, P2RY13, ADORA1, P2RX3. Based on the optimal cut-off value (-0.5605245) of 8 PSRGs scores for the ESCC samples, the ESCC patients were separated into a high-PSRGs scoring group containing 28 samples and a low PSRGs scoring group of 52 samples. The K-M curves revealed marked survival differences in the patients between the scoring groups (*p* = 0.0388), and the probability and duration of survival were lower in the high-PSRGs scoring group (Figure 1C). Further, the 865 DEGs2 were acquired between scoring groups, which contained 386 upregulated and 479 downregulated genes (Figures 1D,E). The above DEGs were overlapped to gain 406 intersection genes (Figure 1F).

### 3.2 The 58 candidate genes involved in multiple pathways and strong interactions between them

**FIGURE 2:**
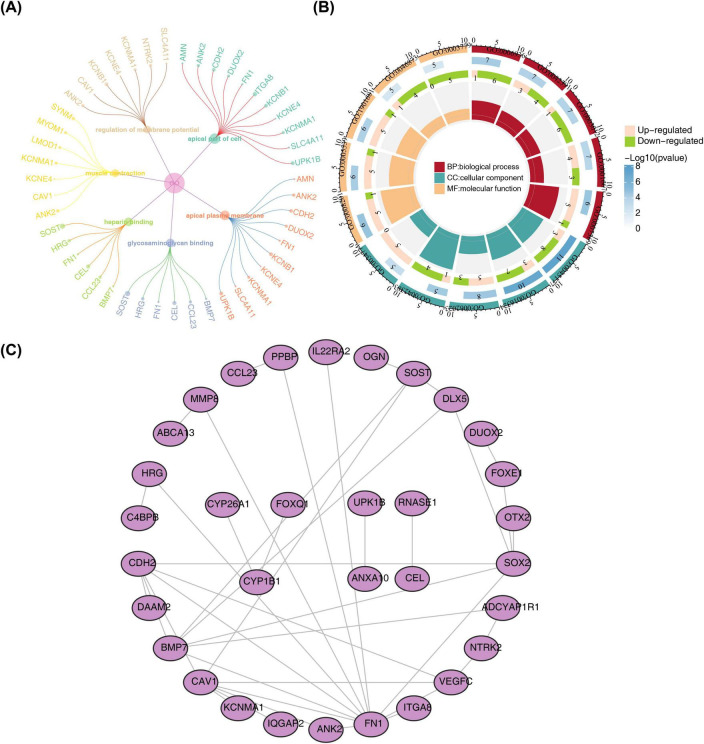
Biological function analysis of 58 candidate genes. KEGG pathway enrichment analysis **(A)**, GO enrichment analysis **(B)** and PPI network construction **(C)** of 58 candidate genes.

To obtain the genes in the intersection genes related to ESCC chemotherapy sensitivity, Wilcoxon was employed to detect the differential expression of the intersection genes in GSE45670 (*p* < 0.05), of which 58 genes exhibited significant expression differences between the sensitivity and insensitivity to CRT treatment and served as candidate genes (Supplementary Figure S1). Further, GO items revealed that these genes were primarily linked to regulation of membrane potential, ameboidal-type cell migration, heparin binding and so on (Figure 2A). KEGG pathway displayed that they were involved in focal adhesion, PI3K-Akt signaling pathway, regulation of actin cytoskeleton (Figure 2B). Additionally, PPI network containing 41 nodes and 40 edges was constructed, which had an average node degree of 1.38, a local clustering coefficient of 0.356 and an enrichment *p*-value of 3.71 × 10^–7^ (Figure 2C).

### 3.3 Constructed an effective and novel risk model for the ESCC

**FIGURE 3:**
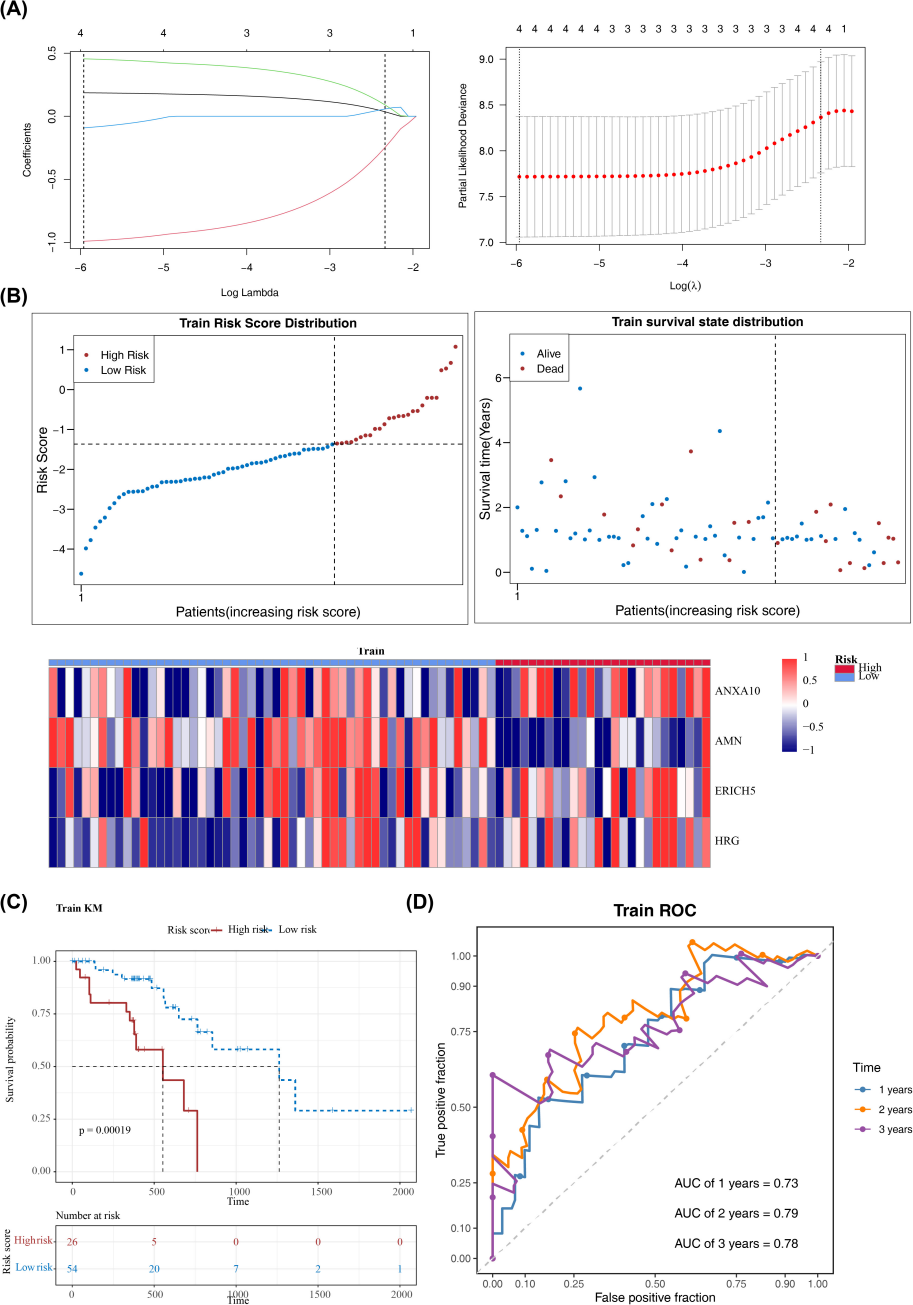
Construction and evaluation of risk model in TCGA-ESCC. **(A)** LASSO regression to screen the prognostic signature genes. **(B)** The survival analysis, risk score distribution curves and heatmaps. Survival analysis comparing the survival rates between patients in low- and high-risk groups; Risk score distribution curves clearly distinguished between the low-risk and high-risk categories; Heatmaps depict the expression levels of prognostic signature genes in the model, highlighting differences in expression between the low- and high-risk groups. Finally, **(C,D)** Kaplan-Meier survival curves and AUC values, which further highlight the disparity in survival outcomes between the two risk groups.

**FIGURE 4:**
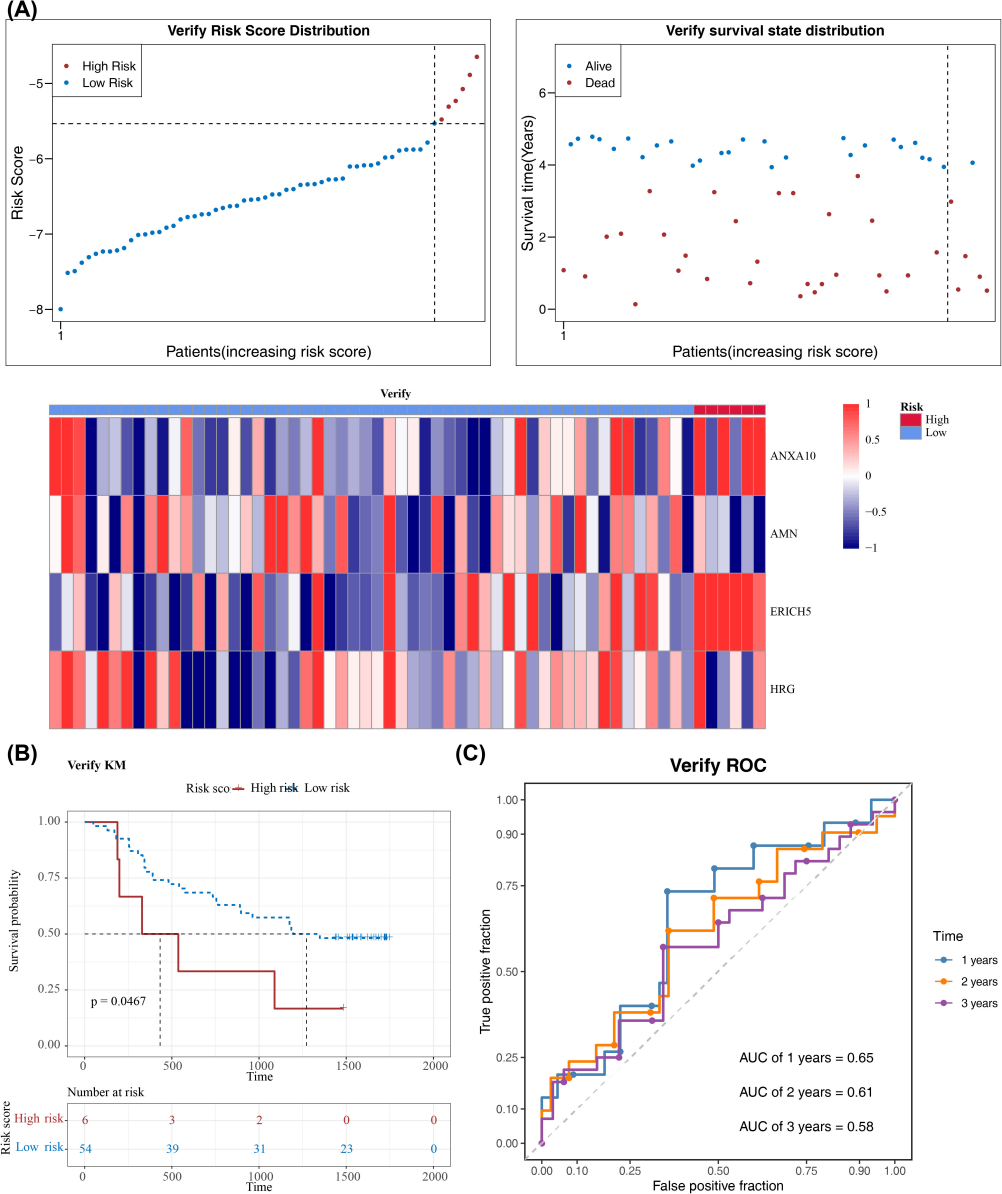
Validation of the risk model in the GSE53622 cohort. **(A)** The distribution of risk scores, associated survival status, and a heatmap displaying the expression of prognostic signature genes in low- and high-risk groups. **(B)** Kaplan-Meier survival analysis comparing overall survival between the two risk groups. **(C)** Time-dependent receiver operating characteristic (ROC) curves at 1, 2, and 3 years, demonstrating the predictive accuracy of the model.

A series of regression analyses were employed to screen the prognostic signature genes of ESCC, resulting in the identification of ANXA10, AMN, ERICH5, and HRG. A novel risk model was then constructed based on these genes (Tables 2, 3 and Figure 3A). Among these, ANXA10, ERICH5, and HRG were associated with increased risk (HR > 1), whereas AMN was associated with decreased risk (HR < 1). Riskscore = 0.1863*ANXA10 + (−0.9909)*AMN + 0.4556*ERICH5 + (−0.092)*HRG. The weights (coefficient signs and magnitudes) of each gene in the final risk score were determined via LASSO-Cox regression, with their direction indicating their conditional contribution to prognosis within the integrated model. In TCGA-ESCC dataset, patients were categorized into high (26 samples) and low (54 samples) risk cohorts based on best cut-off value of the risk score (-1.374722). The risk curves demonstrated increasing risk scores from left to right and an increase in deaths with increasing risk scores (Figure 3B). The K-M curves showed remarkable survival differences in patients between risk cohorts, moreover, the probability of survival was lower for patients in the high-risk cohort (*p* = 0.00019) (Figure 3C). The AUC values for patients at 1-, 2-, 3-years were all > 0.7, demonstrating the accuracy of the model’s predictions (Figure 3D). To perform internal validation and correct for potential overfitting, optimism correction was applied to the model’s performance using 1,000 bootstrap resamples. The results showed a decrease in AUC at 1 year (before correction: 0.73, after correction: 0.63) and 2 years (0.79–0.63), indicating that the original model may have been overfitted and that the corrected performance estimates are more robust. In contrast, the AUC at 3 years increased slightly (0.78–0.80), suggesting that the model retains good discriminative ability in long-term prognostic prediction. In summary, the C-index and AUC obtained after bootstrap correction can serve as more reliable internal performance evaluation metrics for the model (Supplementary Figure S2 and Supplementary Table S1). Further, the validity and accuracy of the model was validated using the same method in GSE53622 (Figures 4A–C). Moreover, in TCGA-ESCC dataset and GSE53622, ANXA10, ERICH5, and HRG were more highly expressed in the high-risk cohort, and AMN was more highly expressed in the low-risk cohort (Figures 3B, 4A).

**TABLE 2:** Univariate Cox regression analysis for prognosis-related genes in patients with ESCC.

	*P*-value	HR (95% CI for HR)
ANXA10	0.0479	1.184 (1.002–1.4)
AMN	0.0116	0.5405 (0.3352–0.8716)
ERICH5	0.0528	1.355 (0.9963–1.842)
HRG	0.0194	2.618 (1.168–5.869)

**TABLE 3:** A proportional hazards assumption test was performed on the prognosis-related genes included in the univariate Cox model.

	Chisq	df	*P*
ANXA10	0.143214	1	0.705106
AMN	0.011787	1	0.913544
ERICH5	0.87482	1	0.349624
HRG	0.011692	1	0.913894

### 3.4 Constructed an effective nomogram based on prognostic signature genes

**FIGURE 5:**
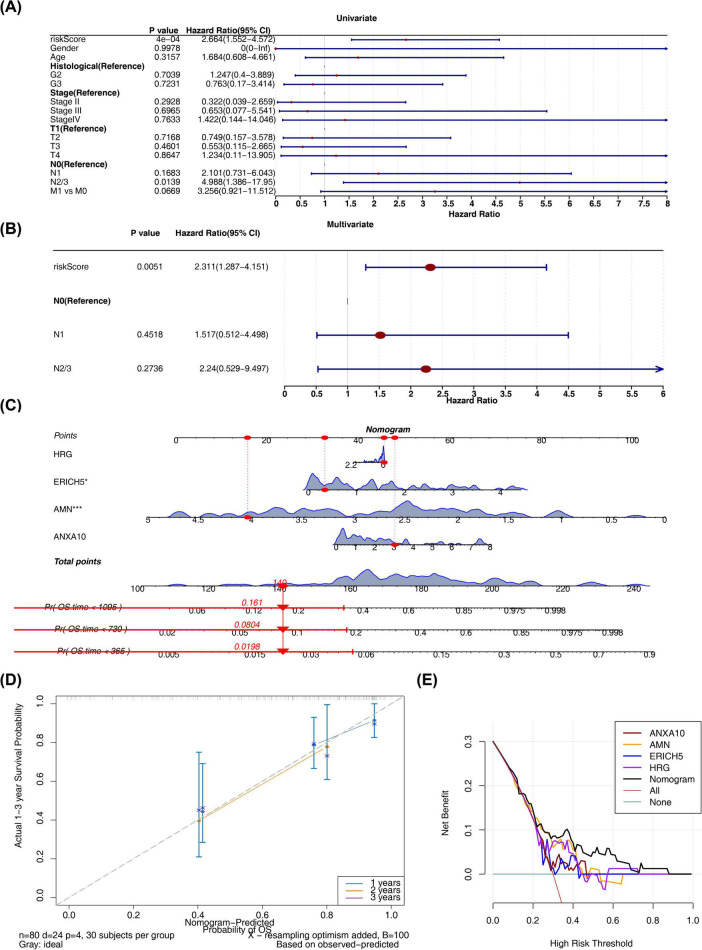
Correlation between the risk score and clinical characteristics. The figure shows **(A)** A univariate Cox regression study of clinical factors and patient risk scores, **(B)** A multivariate Cox analysis of clinical factors and patient risk scores, **(C)** a nomogram for the ESCC cohort that predicts 1-, 3-, and 5-year prognoses, **(D)** Calibration plots of the nomogram for 1-, 3-, and 5-year survival, and **(E)** Using decision curve analysis (DCA) decision curve analysis to assess gene utility for outcomes.

In TCGA-ESCC dataset, there was a remarkable difference in risk scores in histological staging (Supplementary Figure S3). And ERICH5 was remarkably different between subgroups in N stage (Supplementary Figure S4). In addition, survival differences in different clinical features were explored and found to be remarkable between subgroups only for gender (*p* = 0.016) (Supplementary Figure S5). Moreover, there were notable differences in risk cohort patients in the age ≤ 60, G1, and G2 subgroups (Supplementary Figure S6). Further we tested whether the risk score could predict the disease independently of other clinical characteristics and found by regression analysis that only the risk score had a *p* < 0.05, which could be considered as an independent prognostic factor (Figures 5A,B and Table 4). Subsequently, nomogram containing the prognostic signature genes were constructed to predict the 1–3 year survival probability of patients (Figure 5C). Moreover, the predicted probability of patients’ 1-, 2-, 3-year survival in the calibration curve was largely coincident with the ideal curve (Figure 5D), and the overall benefit rates of nomogram in the decision curves were all higher than those of the factors alone (Figure 5E), which comprehensively illustrated the predictive ability of the model with a certain degree of accuracy and validity.

**TABLE 4:** Following single-factor screening, perform a proportional hazards assumption test on the risk score and N.stage.

	Chisq	Df	*P*
Riskscore	0.193813	1	0.659762
N.stage	0.697995	2	0.705395

### 3.5 Functions of different risk cohorts and single-cell functions of prognostic genes

**FIGURE 6:**
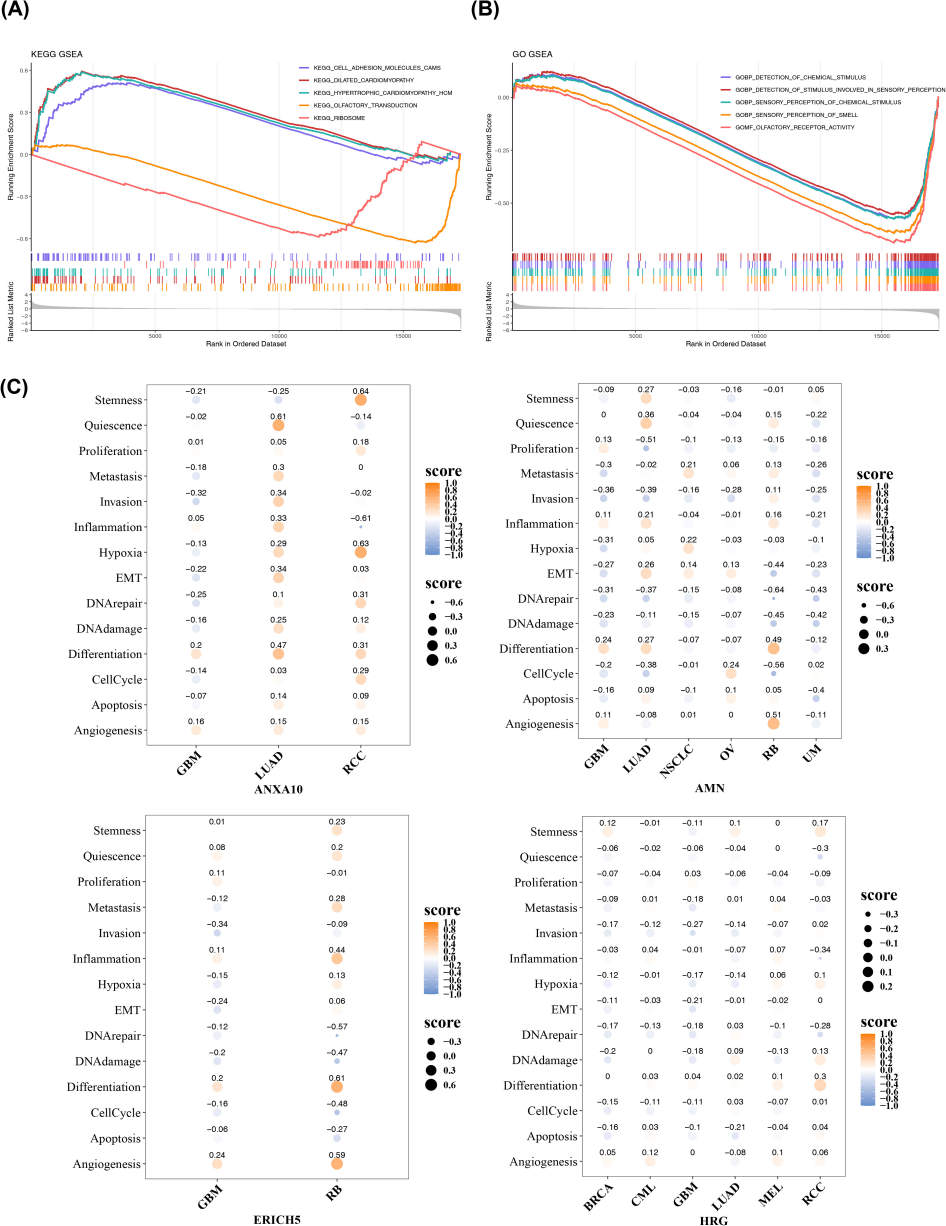
Comparison of molecular mechanisms in different risk cohorts of patients and the single-cell functions of the prognostic signature genes were explored. **(A,B)** GSEA analysis of the different risk cohorts of patients. **(C)** Single-cell functions of prognostic genes in different tumors.

We probed the pathways and functions associated with patients in the risk cohort using GSEA, KEGG pathway displayed that 20 pathways were notably enriched, with the top 5 pathways being cell adhesion molecules, dilated cardiomyopathy, hypertrophic cardiomyopathy, olfactory transduction, and ribosome (Figure 6A). The GO items showed that they were remarkably enriched to 220 pathways and functions containing detection of chemical stimulus, detection of stimulus involved in sensory perception, sensory perception of chemical stimulus, sensory perception of smell and olfactory receptor activity (Figure 6B). Further, the single-cell functions of the prognostic signature genes were explored. Among them, 14 tumor-associated cellular functions contained angiogenesis, apoptosis, cell cycle, differentiation, DNA damage, DNA repair, epithelial mesenchymal transformation (EMT), hypoxia, inflammation, invasion, metastasis, proliferation, quiescence, stemness. In particular, all of these prognostic signature genes were expressed in glioblastoma (GBM), and, ANXA10, AMN and HRG were also expressed in lung adenocarcinoma (LUAD) (Figure 6C). Previous studies have indicated that in LUAD, the transcription factor E2F1 can upregulate ALDH1A1 expression by modulating autophagy processes, thereby enhancing the self-renewal and drug resistance capabilities of lung cancer stem cells (30). The ESCC-signature genes identified in our study were also expressed in LUAD, indicating their potential involvement in similar, conserved pathways related to stemness or therapy resistance, which warrants further investigation.

### 3.6 Possible mutations in ANXA10 and the association between AMN and the immune microenvironment of ESCC

**FIGURE 7:**
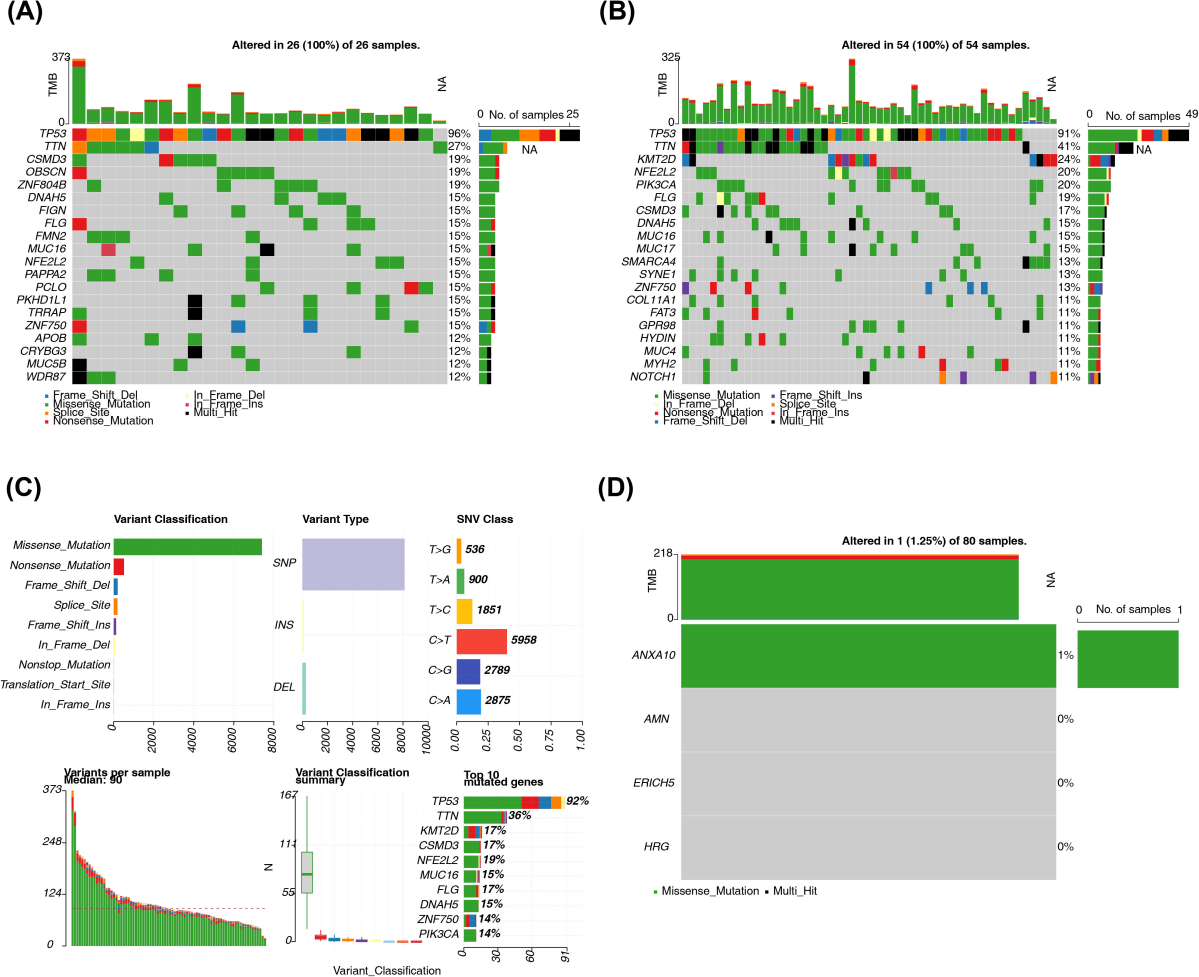
Analysis of somatic mutation information in low- and high-risk groups. Waterfall plot of mutation analysis in high- **(A)** and low-risk **(B)** groups. **(C)** Mutation profiles of the gene in ESCC. **(D)** Mutation analysis of prognostic genes.

**FIGURE 8:**
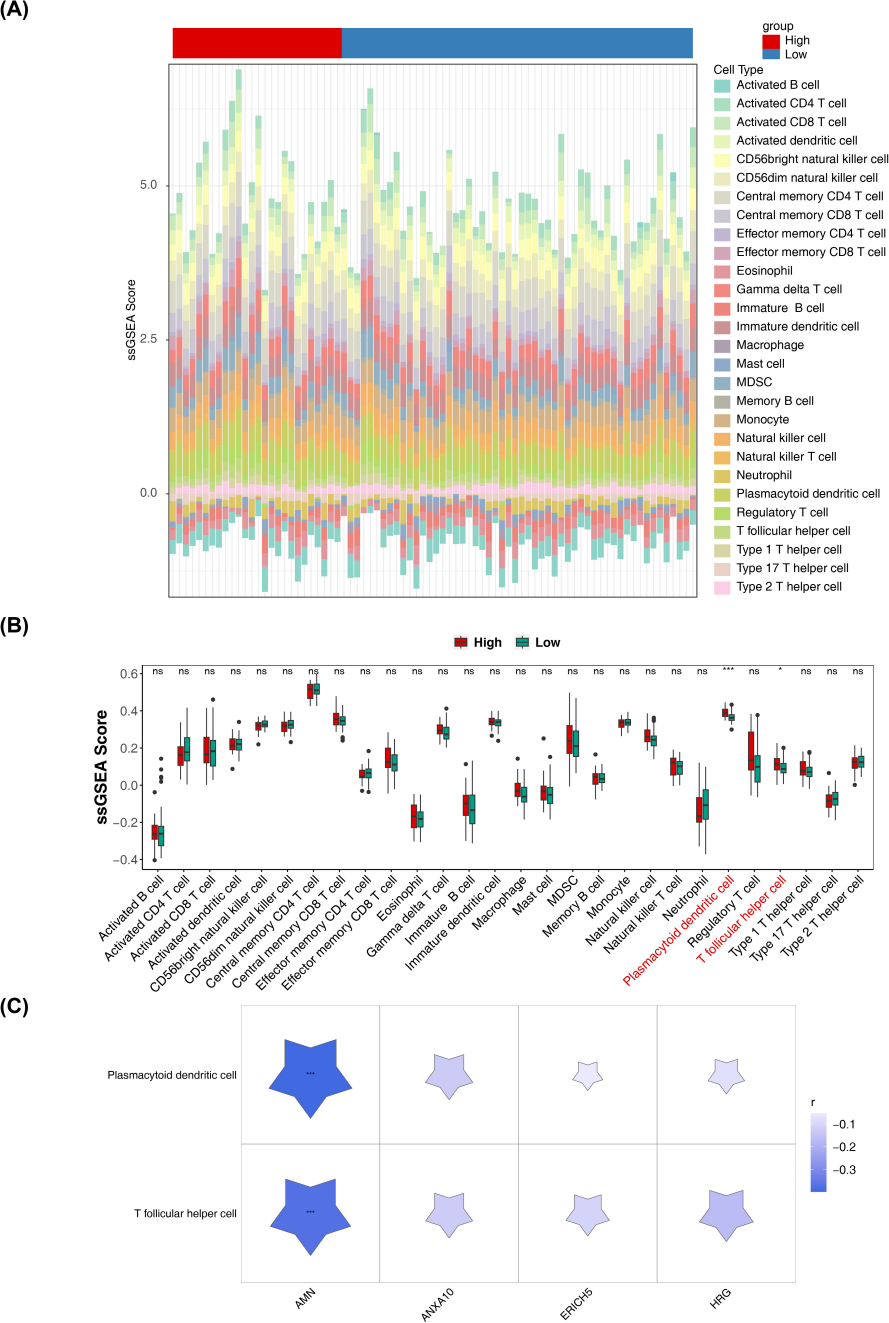
Immune cell infiltration in low- and high-risk groups. **(A,B)** Comparison of 28 immune cells in the low- and high-risk groups. **(C)** Heatmap of prognostic genes correlating with differential immune cells. *p < 0.05 and ***p < 0.001.

Among the different risk cohorts, TP53 had the highest mutation frequency (high: 96%; low: 91%) (Figures 7A,B), with the most likely type of mutation being missense mutation (Figure 7C). Additionally, ANXA10 was likely to have missense mutation (Figure 7D). In addition, exploring the tumor microenvironment of ESCC, the immune score, stromal score, and estimate score were higher in the high-risk cohort (Supplementary Figure S7A), moreover, there may be a positive correlation between the risk score and all of these scores (Supplementary Figure S7B). Further, the immune cell infiltration differences in the risk cohorts were explored, and Figure 8A demonstrates the proportion of 28 immune cells infiltrated in the different risk cohorts. Notably, the high-risk group showed a higher inferred infiltration of plasmacytoid dendritic cells and T follicular helper cells (*p* < 0.05) (Figure 8B). AMN expression showed a markedly negative correlation with the inferred infiltration levels of these immune cells, suggesting a potential association between low AMN expression and an altered immune microenvironment in high-risk ESCC (Figure 8C). It should be noted that these results were derived from computational predictions based on gene expression profiles, and the actual cellular states and functional characteristics still require further validation through experimental techniques such as flow cytometry or spatial transcriptomics.

### 3.7 Drug sensitivity, molecular network, and methylation analysis of prognostic signature genes

**FIGURE 9:**
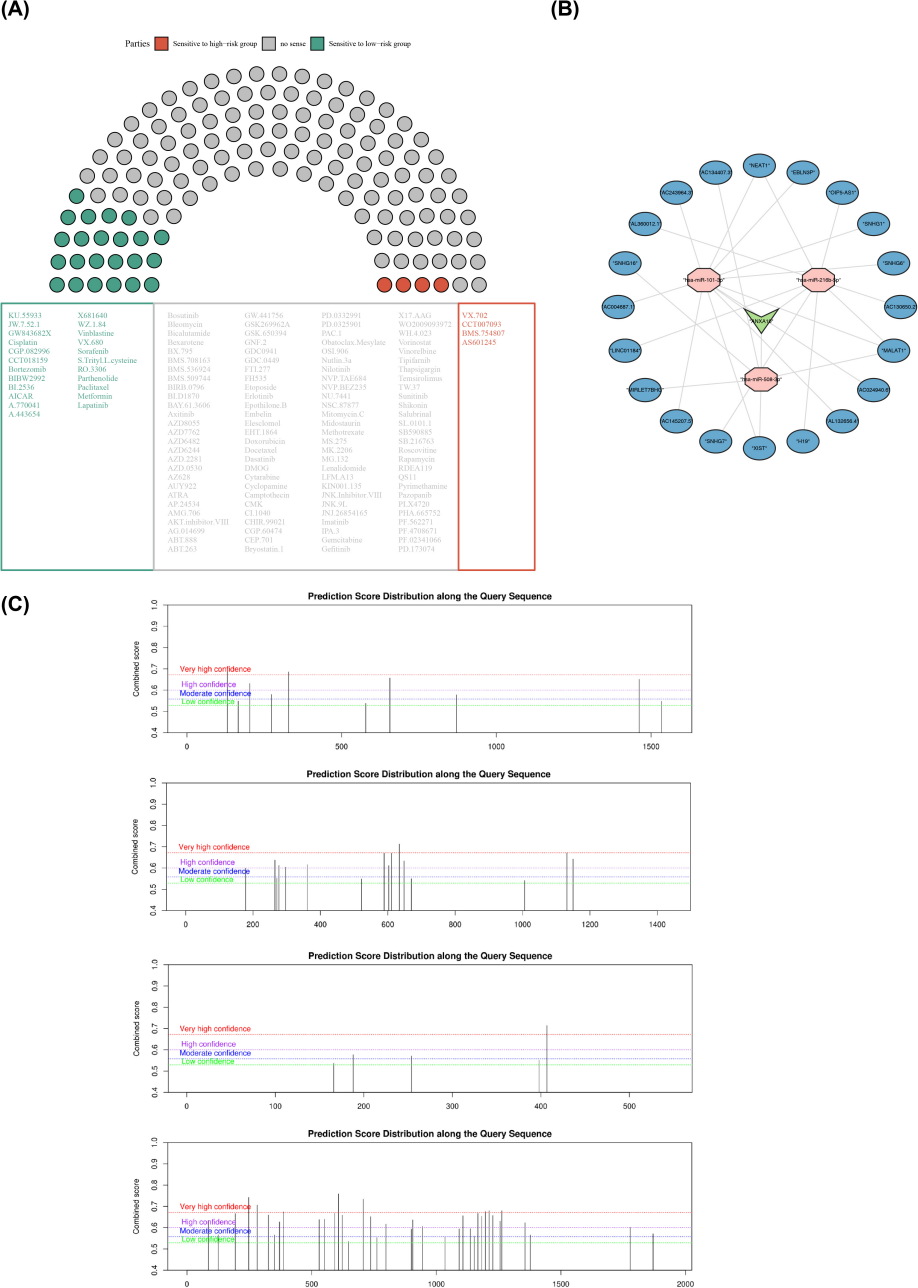
Predictive values in drug sensitivity, molecular network and methylation analysis. **(A)** Box plot presentation of differences in IC_50_ of 138 chemotherapeutic drugs for lung cancer between the low- and high-risk groups, **(B)** probing the lncRNA-mRNA-miRNA regulatory network, **(C)** the m6A modification site of prognostic genes.

This study utilized the GDSC database to conduct predictive analyses of *in vitro* sensitivity to 138 chemotherapeutic/targeted therapies in ESCC. Results revealed significant differences in predicted IC_50_ values for 27 drugs across distinct risk cohorts. Specifically, the gene expression profile of high-risk cohort patients correlated with lower predicted IC_50_ values for drugs VX.702, CCT007093, BMS.754807, and AS601245, suggesting that tumors in this group may exhibit greater *in vitro* sensitivity to these agents at the computational model level (*P* < 0.05). Conversely, lower predicted IC_50_ values for drugs such as sorafenib, metformin, and parthenolide were associated with the low-risk cohort, suggesting that tumors in this group may exhibit greater sensitivity to these agents (Figure 9A). It should be noted that the drug sensitivity predictions in this study were derived entirely from *in vitro* experimental data of single-agent treatment in cancer cell lines obtained from public databases. Future validation through experiments in models (such as patient-derived organoids and *in vivo* models of combination therapies) more closely approximating clinical settings will be required to assess their true therapeutic potential.

In addition, 5 target miRNAs were jointly predicted in miRDB and microcosm databases, namely hsa-miR-508-3p, hsa-miR-216b-5p, hsa-miR-101-3p, hsa-miR-606, and hsa-miR-877-3p. Further, 20 lncRNAs were predicted in Starbase database, containing AC024940.6, SNHG6, EBLN3P, AC243964.3, AC004687.1, AC145207.5, XIST, AL132656.4, MALAT1, SNHG1, NEAT1, AL360012.1, LINC01184, SNHG7, H19, AC130650.2, OIP5-AS1, AC134407.3, SNHG16, MIRLET7BHG. Subsequently, a lncRNA-miRNA-mRNA network was constructed containing ANXA10, 3 miRNAs and 20 lncRNAs (Figure 9B). Moreover, the m6A sites of the prognostic signature genes were analyzed, with ANXA10, AMN, ERICH5 and HRG being susceptible to m6A methylation around 600, 0–500, 400, and 0–1,500 bp, respectively (Figure 9C).

### 3.8 Probed expression of prognostic signature genes in pan-cancer and ESCC

**FIGURE 10:**
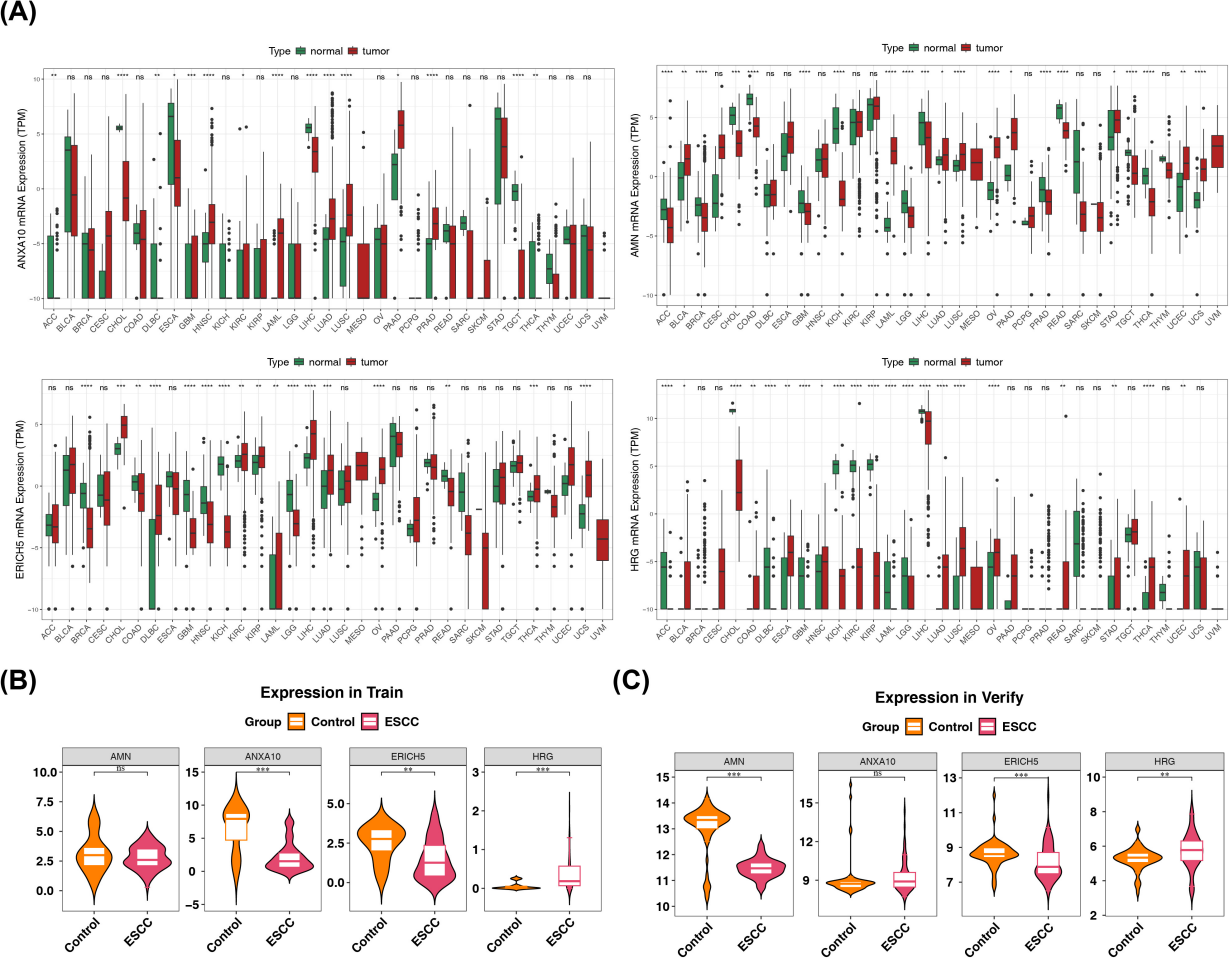
Expression analysis of prognostic genes in pan-cancer and ESCC. Figure represents **(A)** differential TCGA expression in pan-cancer, **(B,C)** The training and verifying cohorts were specifically designed to examine the expression of prognostic genes in ESCC. *p < 0.05, **p < 0.01, ***p < 0.001 and ****p < 0.0001.

The expression of the prognostic signature genes in pan-cancers was understood. ANXA10, AMN, ERICH5, and HRG were remarkably expressed in 15, 21, 17, and 21 tumors, respectively. ANXA10 was markedly down-regulated in ESCA, whereas HRG was notably up-regulated (*p* < 0.05) (Figure 10A). Expression profiles of the prognostic signature genes were extracted at TCGA-ESCC dataset and GSE53622, respectively, where AMN and ERICH5 were markedly down-regulated in the tumor group, while HRG was remarkably up-regulated (*p* < 0.05) (Figures 10B,C).

### 3.9 The effects of chemotherapy, radiotherapy and immunotherapy on gene expression in high- and low-risk groups

**FIGURE 11:**
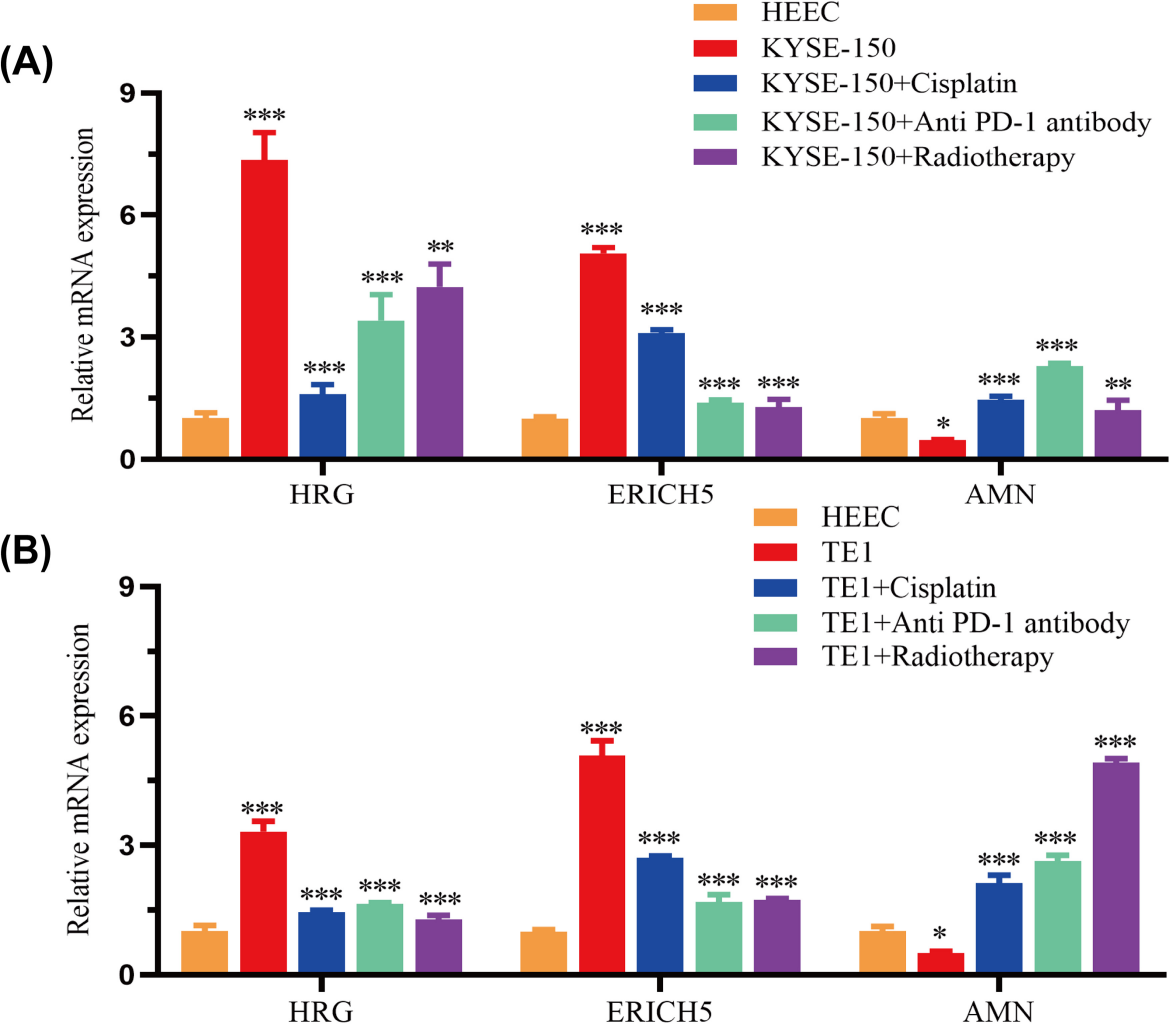
Gene expression in high- and low-risk groups, and validation of chemotherapy, radiotherapy, and immunotherapy. **(A,B)** RT-qPCR analysis of three genes in ESCC cells and HEEC. The effects of cisplatin, an anti-PD-1 antibody and radiotherapy on their expression were also examined. *p < 0.05, **p < 0.01, and ***p < 0.001.

The expression levels of high-risk group genes (HRG and ERICH5) were significantly higher in KYSE-150 and TE1 cells than in HEEC. Conversely, the expression level of the low-risk group gene AMN was lower in ESCC cells than in HEEC. Following treatment with cisplatin or anti-PD-1 antibodies, the expression levels of the HRG and ERICH5 genes decreased significantly, while the expression of the AMN gene increased two- to fivefold. A similar pattern was observed following radiotherapy treatment: high-risk group genes were downregulated, while low-risk genes were upregulated (Figures 11A,B).

## 4 Discussion

ESCC is characterized by rapid tumor growth, high rates of metastasis, and recurrence, which ultimately lead to a poor prognosis for patients (31). There is a pressing need for more accurate prognostic signature genes and models to improve the diagnostic and therapeutic landscape for ESCC patients. PS not only impact the growth, apoptosis, and proliferation of tumor cells but also play a role in recruiting immune cells and regulating immune responses (32). It has been developed as a diagnostic marker or potential treatment for certain solid tumors (33, 34). As a standard treatment modality for advanced ESCC, CRT often yield varying responses among patients due to the heterogeneity of the tumor microenvironment (35). Therefore, it is of utmost importance to further explore the association between PS pathways and the CRT sensitivity of ESCC patients. In this study, bioinformatics methods were utilized to screen four prognostic signature genes (ANXA10, ERICH5, HRG and AMN), explore their biological functions and molecular regulatory mechanisms, and construct a prognostic model. These findings provide new insights into the molecular mechanisms and clinical prognosis of ESCC, laying a hypothetical foundation for subsequent investigations into the functional mechanisms of these genes and their potential therapeutic value.

Annexin A10 (ANXA10), an annexin family member, is a protein-coding gene located on chromosome 4q33 (36). They have numerous biological activities, including apoptosis, calcium signaling, growth control and cell division (37, 38). The overexpression of ANXA10 in ESCC is associated with a poorer prognosis (39). This finding is consistent with the results of our own study, which suggests that ANXA10 may act as an oncogene in the progression of tumors. The potential of ANXA10 as a therapeutic target merits further investigation. Glutamate rich 5 (ERICH5) has been shown to act as a substrate protein for N-myristoyltransferases. It regulates protein stability and protein-protein interactions, which are critical for a variety of biological processes such as cancer progression and immune responses (40). Currently, there has been no in-depth study on the biological mechanism of ERICH5 in ESCC. In this study, the ERICH5 gene was identified for the first time in ESCC, and it may play an important role in the prognosis of ESCC. The results of this study show that ERICH5 is highly expressed in ESCC high-risk group and is associated with poor prognosis, and its role in the prognosis of ESCC remains to be further explored. Histidine-rich glycoprotein (HRG), a heparin-binding plasma protein, has the ability to bind a wide range of ligands involved in the regulation of immunity, cell adhesion, angiogenesis and the activity of degradative enzymes such as plasminogen/plasmin (41). It is possible that HRG may serve as a therapeutic target for pancreatic cancer, making it more sensitive to radiotherapy and chemotherapy (42). Although HRG has been reported to potentially influence radiotherapy sensitivity in pancreatic cancer, this study found its expression to be upregulated in ESCC and associated with high risk, providing clues for further investigation into its role in ESCC treatment response. However, the precise mechanism by which this process occurs remains to be fully elucidated through experimental validation. Amnion associated transmembrane protein (AMN) expression is a significant indicator of disease progression, clinical biological behavior, and prognosis. The study showed that the AMN gene was identified as a prognostic gene for kidney renal clear cell carcinoma (KIRC), providing new ideas for immunotherapy and gene-targeted drugs for KIRC (43). AMN may be used as a potential diagnostic marker to differentiate chronic pancreatitis from pancreatic adenocarcinoma (44). In view of the regulatory function of AMN in other tumors, it is hypothesized that it may also serve as a key tumor suppressor in ESCC. The objective of this study is to furnish preliminary bioinformatics and experimental evidence that will substantiate this hypothesis. In our experiment, cisplatin, immune checkpoint inhibitors (anti-PD-1), and radiotherapy have been found to modulate the expression of three prognostic signature genes (ERICH5, HRG, and AMN), indicating their role in ESCC drug response. AMN exerts a tumor-suppressing effect, and its therapeutic upregulation has the potential to augment sensitivity to chemoradiotherapy and immunotherapy. Nevertheless, the potential mechanisms of these drugs in ESCC patients warrant further investigation.

GSEA analyses were conducted to better understand the mechanisms underlying the features. This study obtained multiple functions and pathways between high and low risk groups, including cell adhesion molecules (CAMs), focal adhesion. CAMs are an umbrella term for several families of molecules that enable cells to adhere to and interact with the extracellular matrix and other cells (45). They are divided into the cadherin family, integrin (ITG) family, selectin family, immunoglobulin superfamily, and various unclassified adhesion molecule groups (46). E-cadherin is an intercellular adhesion protein. It has been found that the abnormal level of expression of E-cadherin plays an important role in the metastatic stage of the ESCC (47). E-cadherin promotes ESCC cell migration and invasion through loss of cell polarity and maintenance of the tumor basal cell state (48). These studies point to the important role of the CAMS-related signaling pathways in ESCC.

This study found that the high-risk group exhibited higher levels of computationally inferred infiltration of plasma cell-like dendritic cells and T follicular helper cells, with AMN expression showing a significant negative correlation with these infiltration levels. Notably, dendritic cells act as key immunomodulatory factors, presenting exogenous antigens to lymphocytes, triggering adaptive immune responses, and thus influencing the mechanisms of tumor immune surveillance and escape (49). A growing body of evidence emphasizes the potential importance of CD4 T follicular helper (Tfh) in cancer and immunotherapy (50). Moreover, CD8 T cells regulate the expression of Tfh cell-associated genes in a TCF1-and Id2-dependent manner to expressing CXCR5 and other Tfh cell-associated genes, and it is these CXCR5 CD8 T cells that are most responsive to PD1 immunotherapy (51). Based on the findings of this study, we hypothesize that reduced AMN expression may be associated with changes to immune microenvironments. However, the specific regulatory mechanisms require validation through further experimental research.

A variety of lncRNA-miRNA-mRNA have been shown to be involved in the regulation of tumorigenesis and progression (52). To explore the function and regulatory mechanism of prognostic genes at a deeper level, we constructed the lncRNA-miRNA-mRNA regulatory network of prognostic genes. We predicted 5 target miRNAs, which are hsa-miR-508-3p, hsa-miR-216b-5p, hsa-miR-101-3p, hsa-miR-606, and hsa-miR-877-3p. Besides, we predicted 20 lncRNAs, namely AC024940.6, SNHG6, EBLN3P, AC243964.3, AC004687.1, AC145207.5, XIST, AL132656.4, MALAT1, SNHG1, NEAT1, AL360012.1, LINC01184, SNHG7, H19, AC130650.2, OIP5-AS1, AC134407.3, SNHG16, MIRLET7BHG. The lncRNA SNHG6 was up-regulated in ESCC tissues, and its expression level was closely associated with patient prognosis, lymph node metastasis, distant metastasis and TNM staging (53). Wu et al. demonstrate that high expression of XIST predicted poor prognosis in patients with ESCC. Further study reveals that upregulation of XIST expression may promote malignant progression of ESCC by regulating the miR-101/EZH2 axis and could be used as a therapeutic target for ESCC (54). Furthermore, SNHG16 was found to be highly expressed in ESCC and intraepithelial neoplasia samples, and its expression level correlated with tumor differentiation and T stage. Mechanistically, the SNHG16-EIF4A3-RhoU signaling pathway is directly involved in the initiation and development of ESCC and may serve as a novel therapeutic target for ESCC (55). In conclusion, lncRNA-miRNA-mRNA represent a more comprehensive intermolecular regulatory mode of RNA. Compared with miRNA regulatory network, lncRNA-miRNA-mRNA regulatory network is more delicate and complex, involving more RNA molecules, which is of great significance for elucidating the mechanism of tumor progression and therapeutic response.

In this study, we screened and analyzed 4 prognostic gene (ANXA10, ERICH5, HRG, and AMN), and further analyzed the prognosis model constructed for prognostic gene. This includes analysis of the biological function, molecular regulatory mechanism, and the association with immune characteristics of genes related to PS and chemoradiotherapy sensitivity in ESCC. Furthermore, preliminary validation of the effects of chemoradiotherapy and immunotherapy on the expression of prognostic genes has been conducted through cellular experimentation. It may provide new ideas for CRT in ESCC. However, it is important to note that this study is subject to the following major limitations: Firstly, the construction and validation of the prognostic model was based exclusively on public databases, and it has not yet undergone validation in a large-scale prospective clinical cohort. It is recommended that future studies provide further validation of the model’s robustness and generalizability in large-sample, multi-center cohorts. Furthermore, artificial intelligence (AI) shows great promise in integrating multi-omics data and identifying characteristics of cancer stem cells (56). Future applications could use AI algorithms to refine this model further, enabling the precise quantification of the ESCC stemness index. This would clarify its prognostic value at a mechanistic level and advance personalized therapies targeting stem cells. Secondly, the key datasets utilized for the screening of CRT-sensitivity-related genes exhibited limited sample sizes, a factor that may have exerted an influence on the statistical power. The prognostic value of these genes will be validated in larger independent cohorts. Thirdly, the present analysis is confined to the transcriptomic level; the expression and clinical significance of the four signature genes have not yet been validated at the protein level. The subsequent phase of the research will entail the validation of protein expression in ESCC clinical samples using immunohistochemistry, Western blot, and other techniques. Fourthly, the specific functional mechanisms of these genes in ESCC remain unclear. Subsequent studies will integrate cellular and animal experiments to elucidate their roles in tumor progression and treatment response.

## 5 Conclusion

This study employed bioinformatics approaches on public datasets and identified four prognostic signature genes—ANXA10, ERICH5, HRG, and AMN—through differential expression analysis. A corresponding ESCC risk model was constructed, demonstrating reliable prognostic predictive power, with the risk score confirmed as an independent prognostic factor. A nomogram based on this model accurately predicted patient survival at 1 and 3 years. Further mechanistic investigation revealed significant enrichment of pathways such as cell adhesion molecules in the high-risk group, along with a potential missense mutation in ANXA10. Increased infiltration of plasmacytoid dendritic cells and follicular helper T cells was observed, which correlated negatively with AMN expression. The high-risk group also exhibited heightened sensitivity to drugs such as VX-702 and BMS-754807, suggesting novel strategies for individualized therapy. In summary, the four genetic features identified in this study provide new insights into understanding the pathogenesis of ESCC. The constructed risk model offers a potential reference framework for clinical prognosis assessment and personalized treatment, as well as directions for further research.

## Data Availability

The original contributions presented in the study are included in the article/Supplementary material, further inquiries can be directed to the corresponding authors.

## Footnotes

1
https://xenabrowser.net/datapages/

2
https://www.ncbi.nlm.nih.gov/geo/

3
https://www.gsea-msigdb.org/gsea/msigdb

4
http://biocc.hrbmu.edu.cn/CancerSEA/

5
http://www.cuilab.cn/sramp/

6
https://www.cancerrxgene.org/

7
https://rnasysu.com/encori/
