# Supplementary figures and images for: Construction of a novel risk model for esophageal squamous cell carcinoma associated with purinergic signaling pathways and chemoradiotherapy sensitivity genes
Source: Front Med (Lausanne). 2026 Mar 17;13:1746281. doi: 10.3389/fmed.2026.1746281 (PMC13036854; doi:10.3389/fmed.2026.1746281)

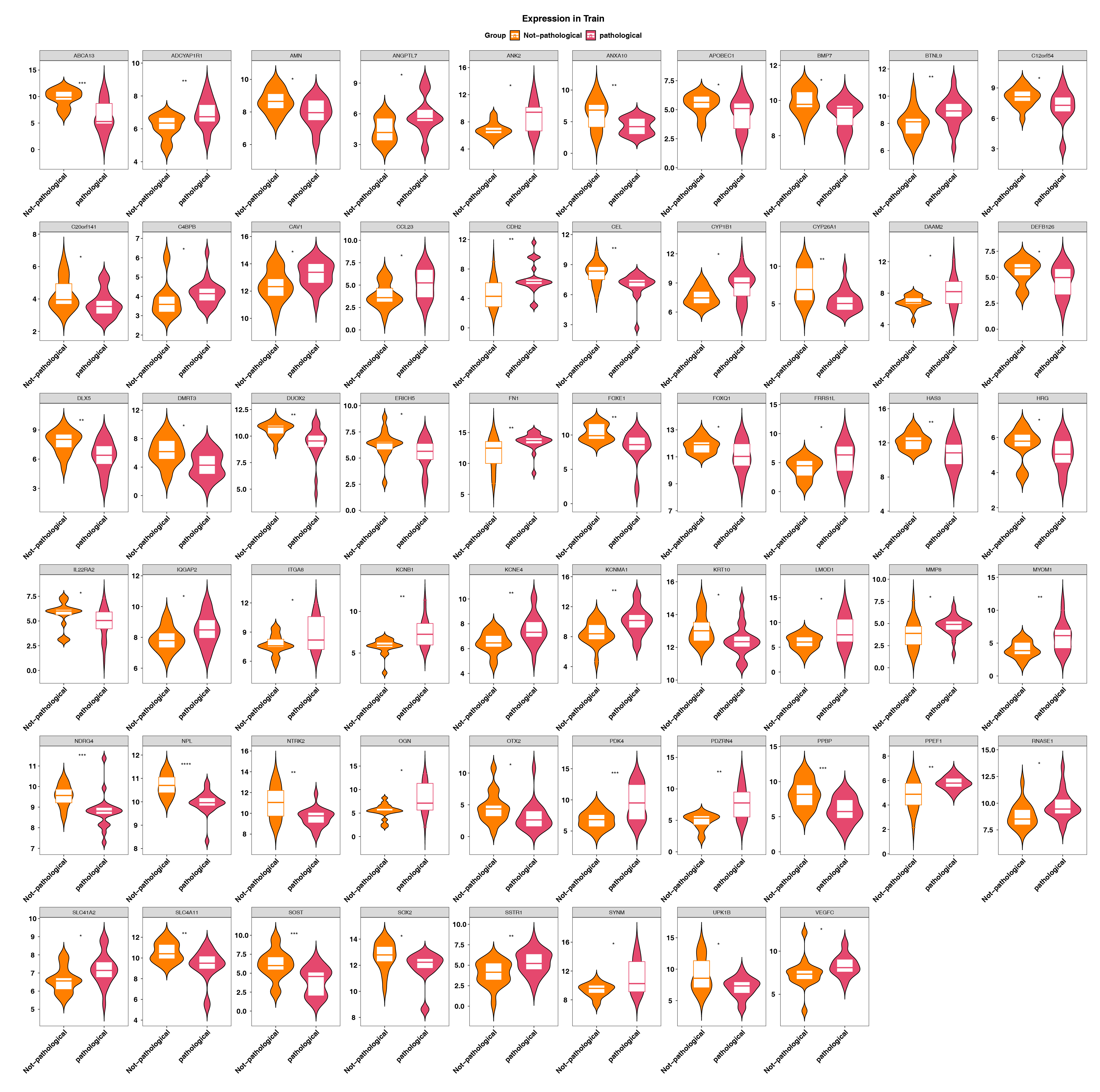

Supplement: Supplementary file 2 [file Image_1.tif]

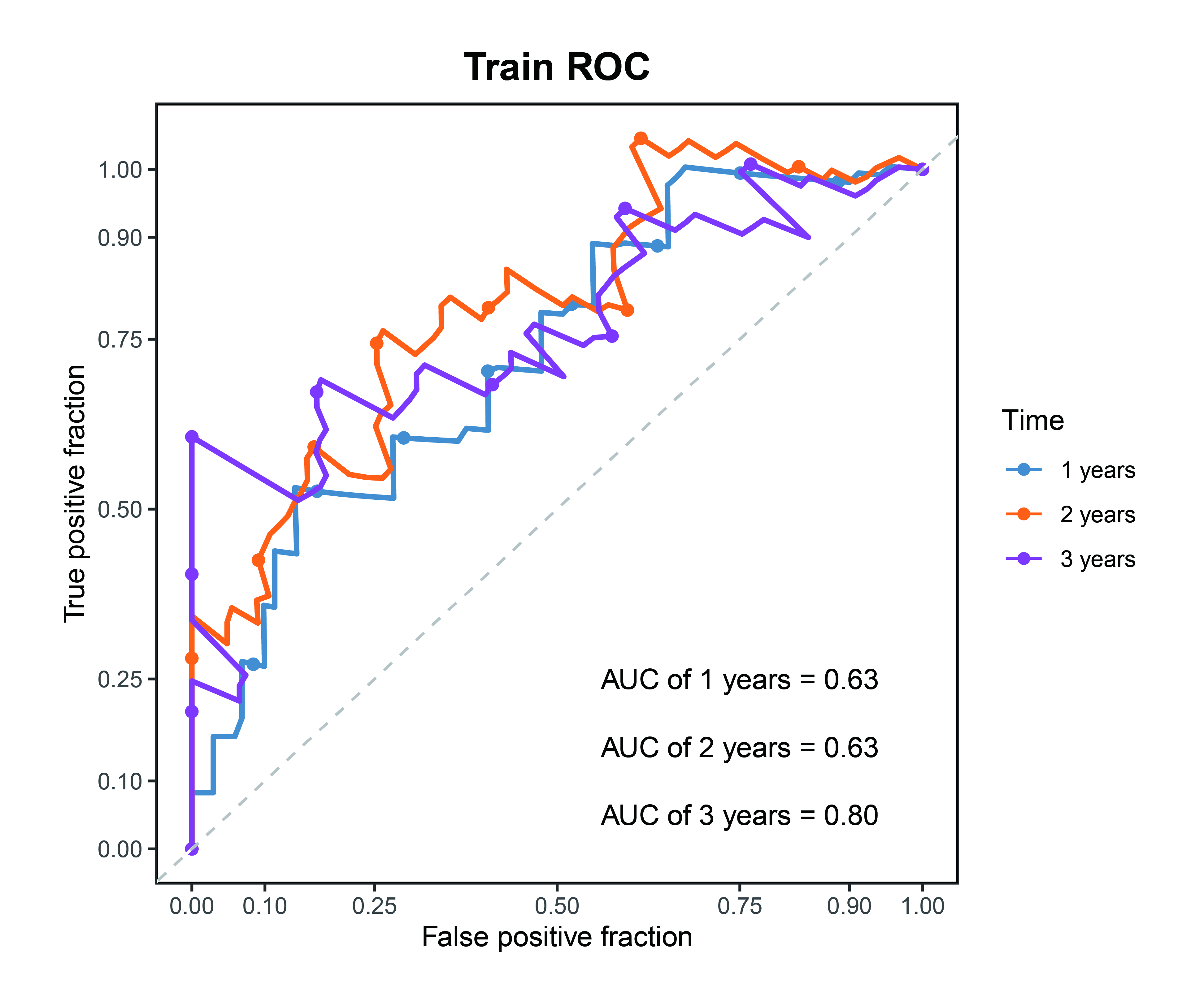

Supplement: Supplementary file 3 [file Image_2.tif]

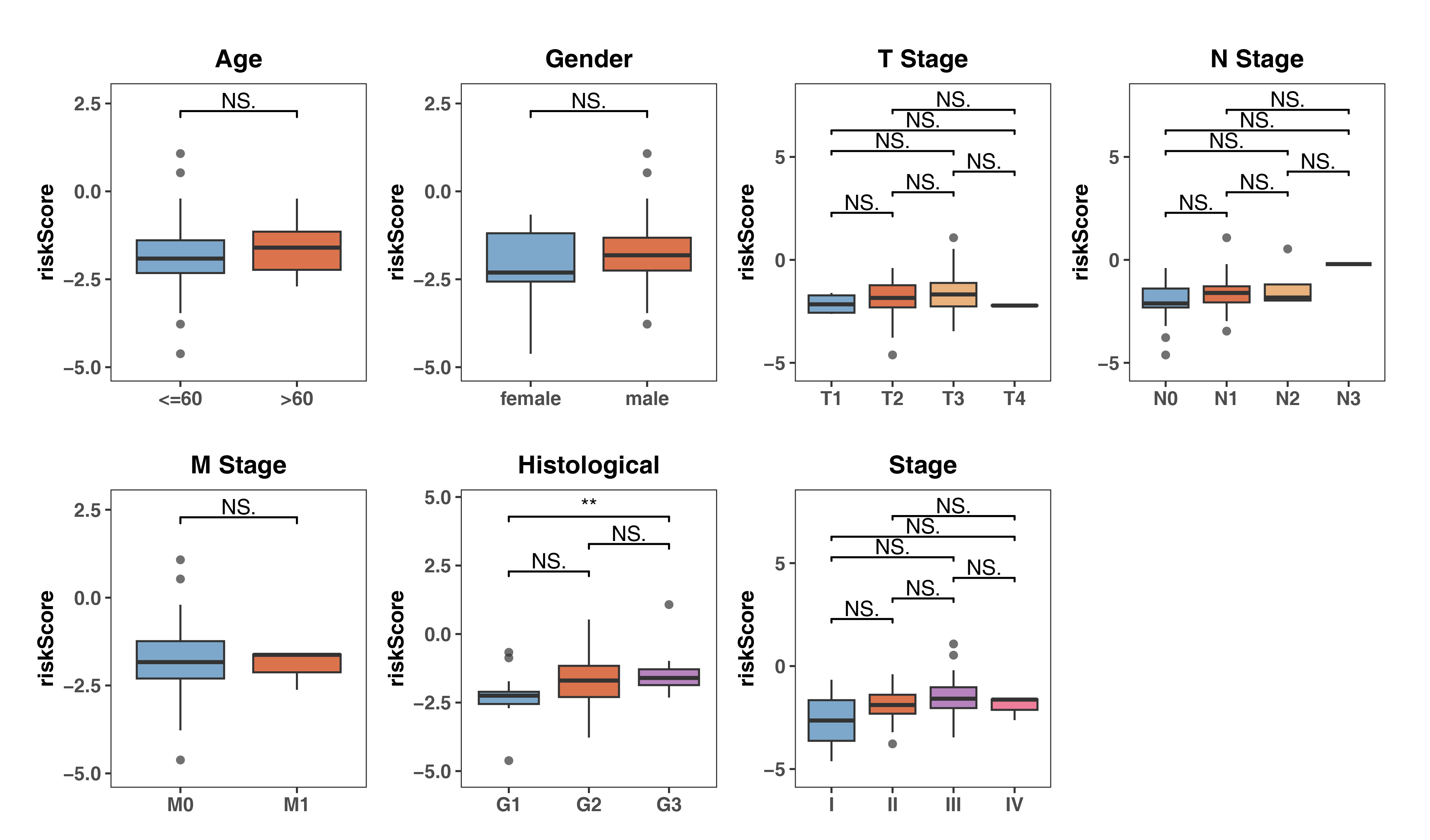

Supplement: Supplementary file 4 [file Image_3.tif]

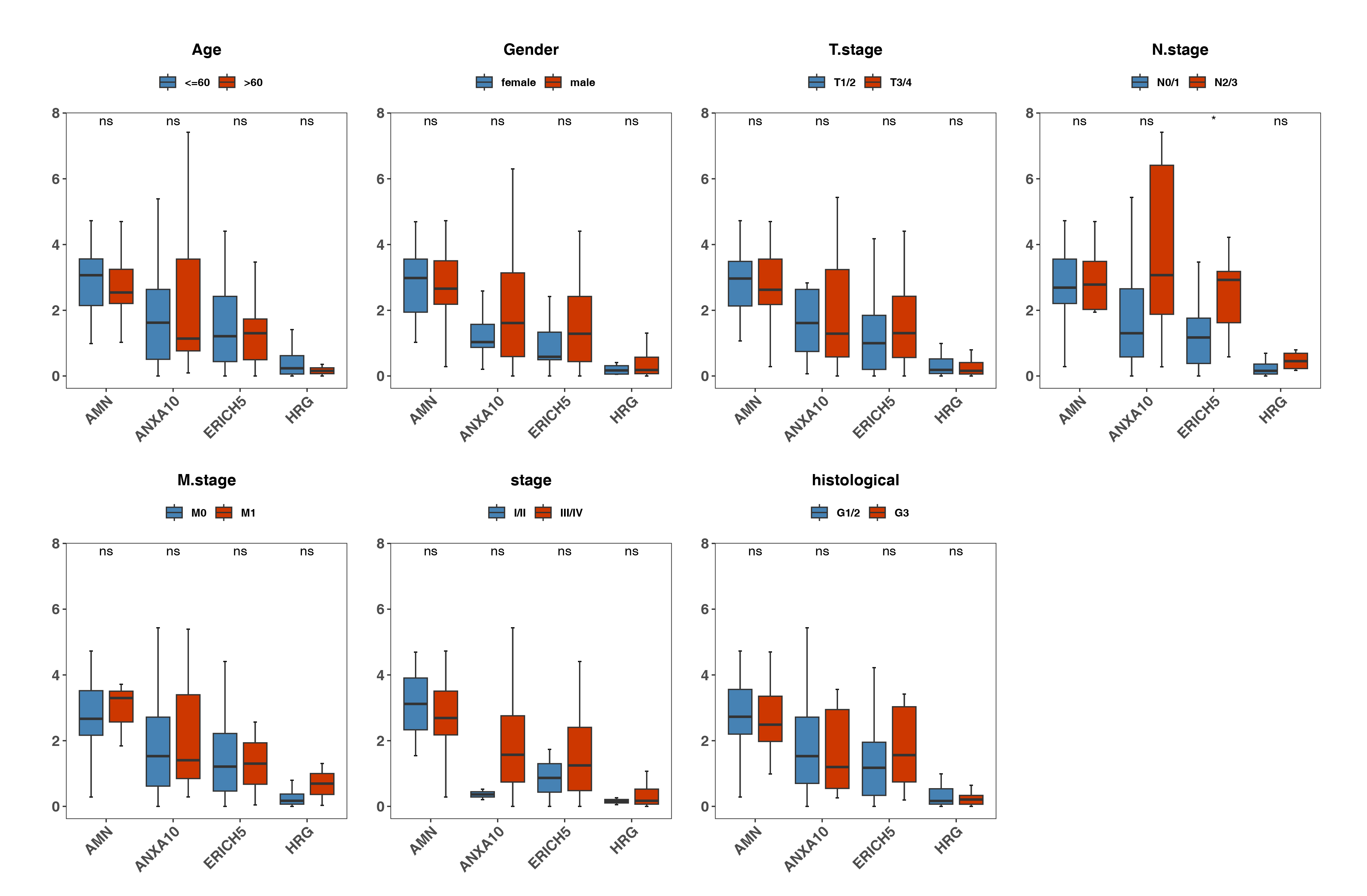

Supplement: Supplementary file 5 [file Image_4.tif]

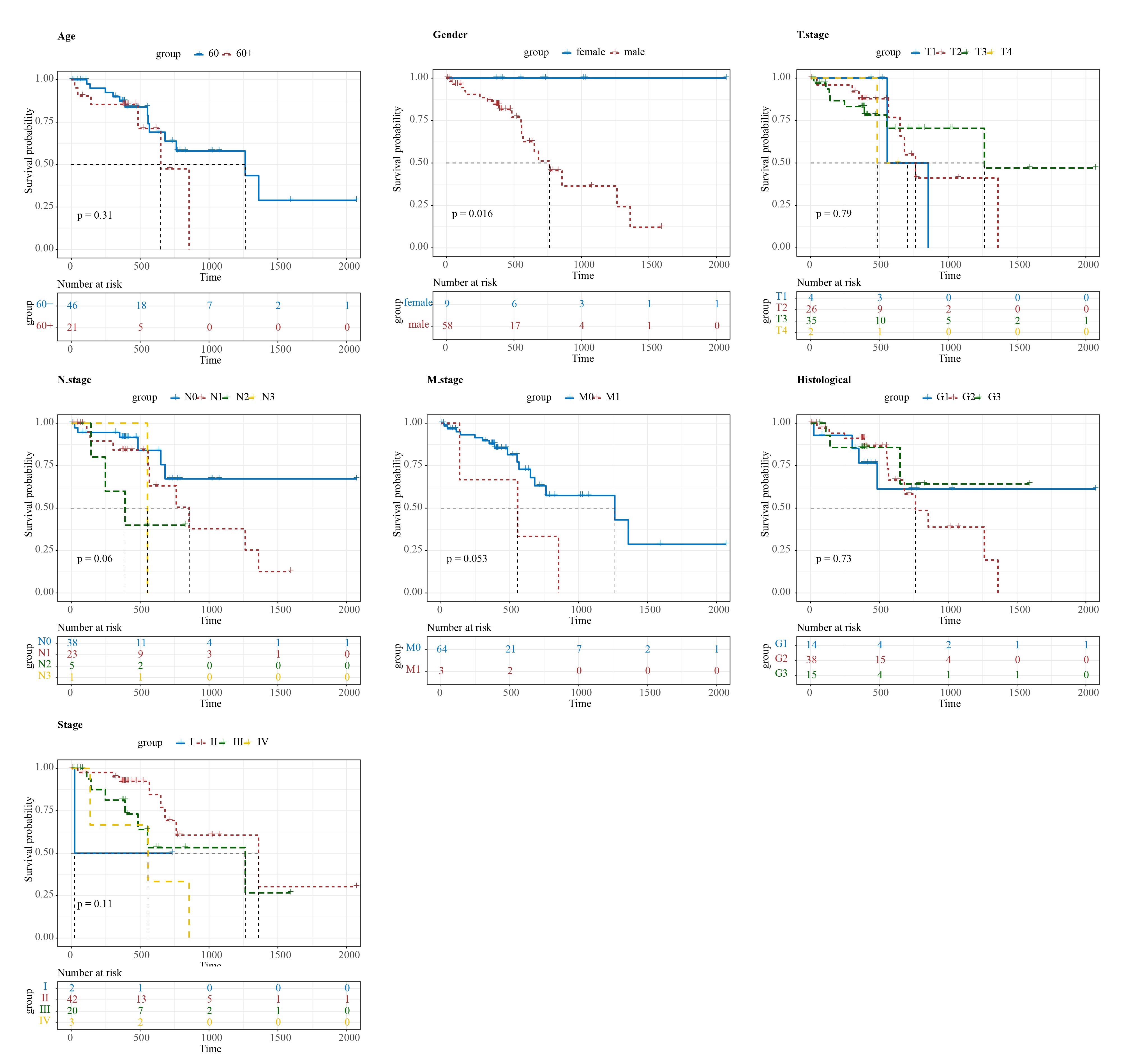

Supplement: Supplementary file 6 [file Image_5.tif]

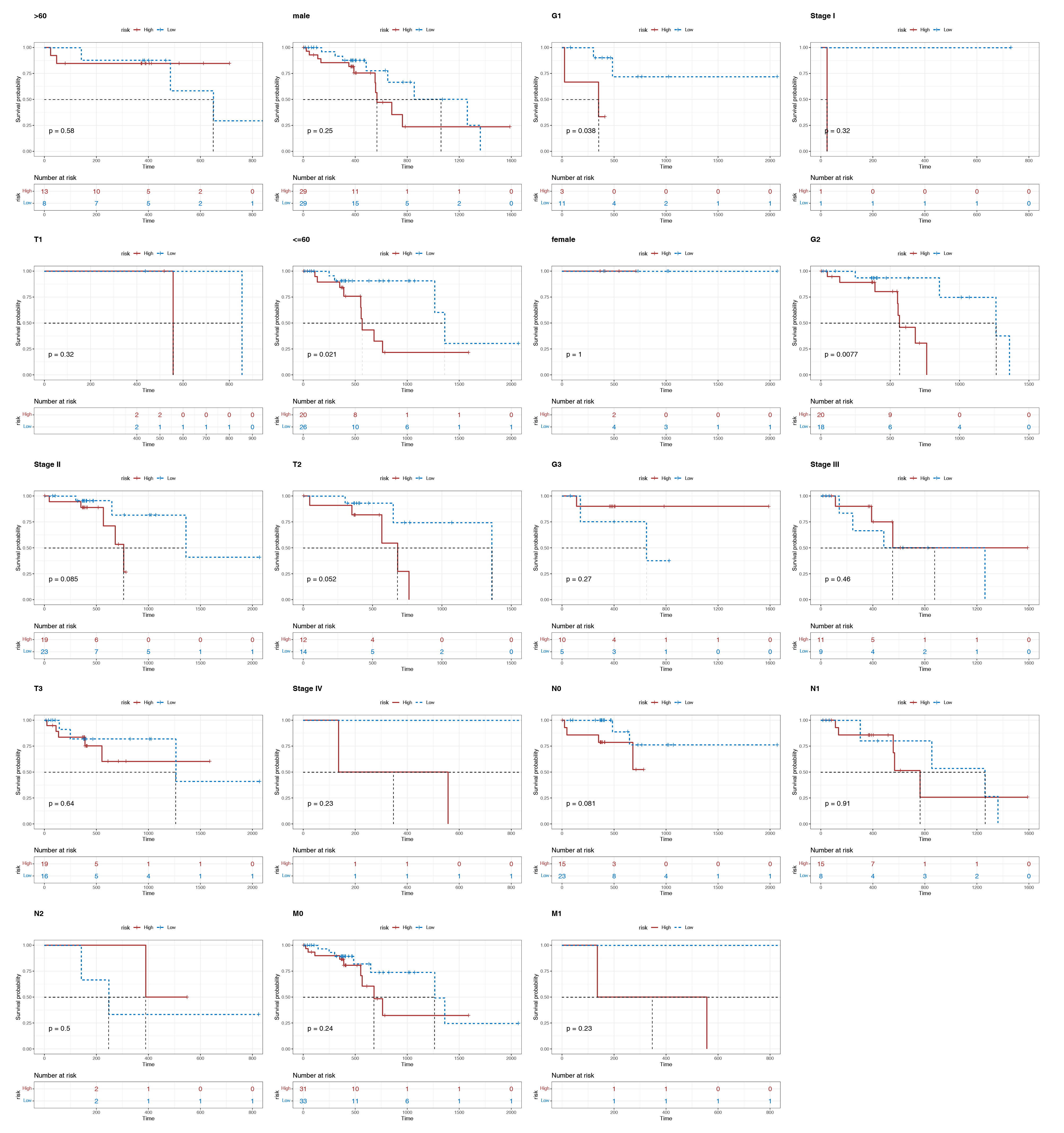

Supplement: Supplementary file 7 [file Image_6.tif]

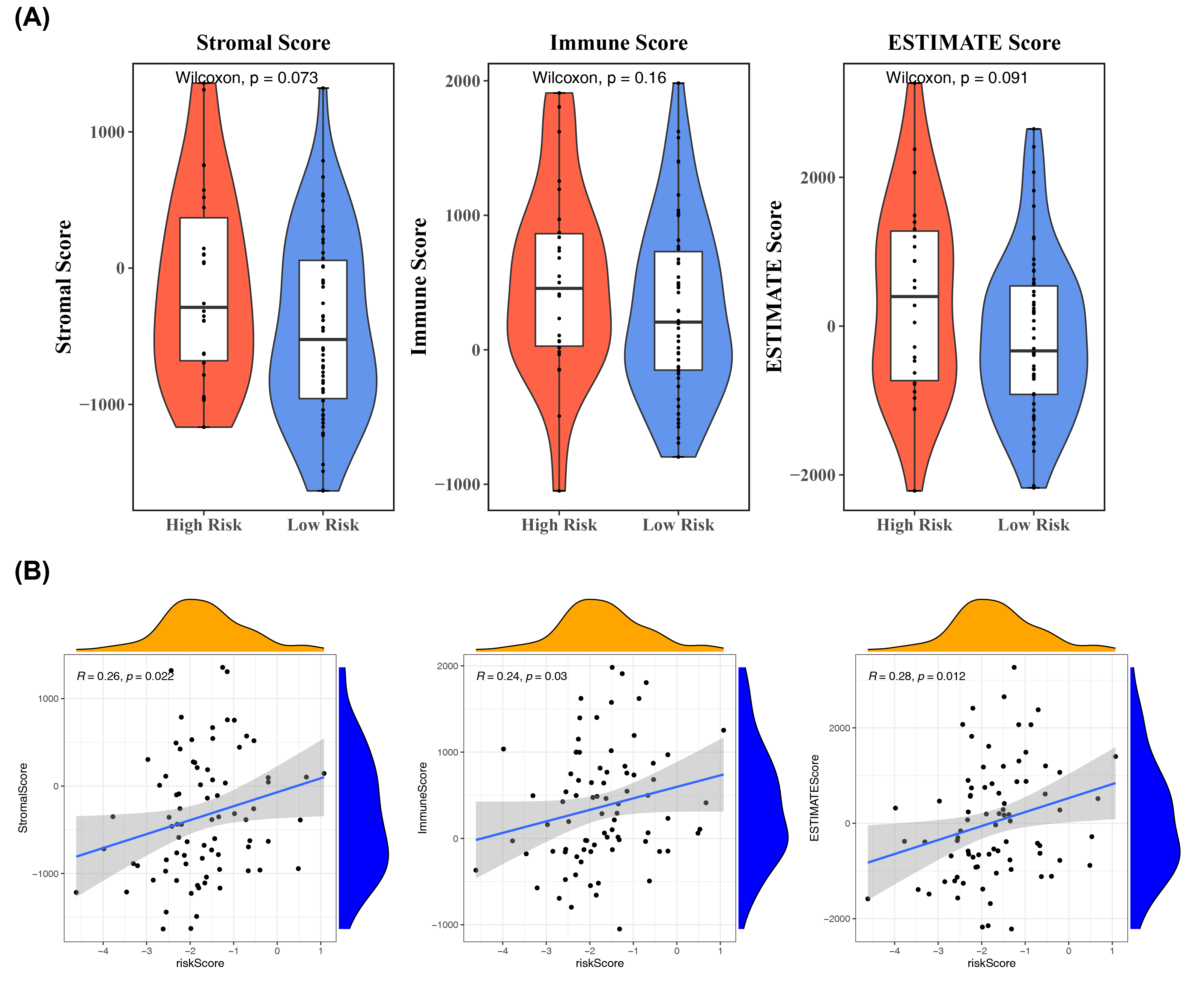

Supplement: Supplementary file 8 [file Image_7.tif]
